# Characterization of the maize lipoxygenase gene family in relation to aflatoxin accumulation resistance

**DOI:** 10.1371/journal.pone.0181265

**Published:** 2017-07-17

**Authors:** Oluwaseun F. Ogunola, Leigh K. Hawkins, Erik Mylroie, Michael V. Kolomiets, Eli Borrego, Juliet D. Tang, W. Paul Williams, Marilyn L. Warburton

**Affiliations:** 1 Department of Plant and Soil Sciences, Mississippi State University, Starkville, MS, United States of America; 2 USDA-ARS Corn Host Plant Resistance Research Unit, Starkville, MS, United States of America; 3 Department of Plant Pathology and Microbiology, Texas A&M University, College Station, Texas, United States of America; 4 USDA FS Forest Products Laboratory, Durability and Wood Protection, Starkville, MS, United States of America; Mahatma Phule Krishi Vidyapeeth College of Agriculture, INDIA

## Abstract

Maize (*Zea mays* L.) is a globally important staple food crop prone to contamination by aflatoxin, a carcinogenic secondary metabolite produced by the fungus *Aspergillus flavus*. An efficient approach to reduce accumulation of aflatoxin is the development of germplasm resistant to colonization and toxin production by *A*. *flavus*. Lipoxygenases (LOXs) are a group of non-heme iron containing dioxygenase enzymes that catalyze oxygenation of polyunsaturated fatty acids (PUFAs). LOX derived oxylipins play critical roles in plant defense against pathogens including *A*. *flavus*. The objectives of this study were to summarize sequence diversity and expression patterns for all LOX genes in the maize genome, and map their effect on aflatoxin accumulation via linkage and association mapping. In total, 13 LOX genes were identified, characterized, and mapped. The sequence of one gene, *ZmLOX10*, is reported from 5 inbred lines. Genes *ZmLOX1*/*2*, *5*, *8*, *9*, *10* and *12* (GRMZM2G156861, or V4 numbers ZM00001D042541 and Zm00001D042540, GRMZM2G102760, GRMZM2G104843, GRMZM2G017616, GRMZM2G015419, and GRMZM2G106748, respectively) fell under previously published QTL in one or more mapping populations and are linked to a measurable reduction of aflatoxin in maize grains. Association mapping results found 28 of the 726 SNPs tested were associated with reduced aflatoxin levels at p ≤ 9.71 x 10^−4^ according to association statistics. These fell within or near nine of the ZmLOX genes. This work confirms the importance of some lipoxygenases for resistance to aflatoxin accumulation and may be used to direct future genetic selection in maize.

## Introduction

*Aspergillus flavus* is a fungus found mostly in soil, but can also be found growing on plant products, especially in oil rich seeds such as corn, cotton and peanuts. *A*. *flavus* produces a secondary metabolite known as aflatoxin, which is a carcinogen, mutagen, and hepatotoxin, harmful to humans, poultry and other farm animals [1]. Most commercial maize hybrids are susceptible to *A*. *flavus* infection, which ultimately leads to high and unsafe levels of aflatoxin under environmental conditions favoring fungal growth and sporulation. Aflatoxin was first discovered and characterized in the early 1960’s when more than 100,000 turkeys in England died after consuming mold contaminated peanut meal [2, 3]. Infection by *A*. *flavus* and *A*. *parasiticus* (which can also produce aflatoxin) can be recognized by gray-green or yellow-green fungal growth on the corn kernels. Why some Aspergillus fungi produce aflatoxin is not well understood, but it has been reported that both *A*. *flavus* growth and the production of aflatoxins is favored by abiotic stress such as drought, high heat and nutrient deficiencies [4]. Aflatoxins can be detected either on corn still in the field or in storage following harvest, where it can continue to accumulate in grain stored under humid conditions. Furthermore, the risk of aflatoxin contamination is higher when the husks or kernels are damaged (commonly by ear worms), which creates an opportunistic entry point for fungal infection.

Development of resistant germplasm is one of the most effective methods to reduce aflatoxin accumulation in maize, but the quantitative nature of the trait and the high environmental variation associated with it make creation of resistant germplasm difficult to achieve. Identification of maize candidate genes that contribute to aflatoxin resistance via QTL or association mapping and subsequent development of linked molecular markers for marker assisted selection (MAS) can speed development of resistant maize varieties. Host plant resistance mechanisms, particularly for resistance to *A*. *flavus*, are slowly being uncovered [4, 5] but many factors have yet to be determined. Nevertheless, maize breeders have been able to develop resistant germplasm using phenotypic selection procedures as reviewed in [6]. Unfortunately, all aflatoxin resistant breeding lines perform sub-optimally in the US Corn Belt, as all derive their resistance from tropical germplasm [7]. Transfer of the quantitative resistance to temperate breeding lines has been slow and incomplete, to date.

When plants come under insect or fungal attack, genetic and metabolic processes are initiated, including the production of defense hormones and other chemical signals to repel or block the attack, and/or emission of herbivore induced plant volatiles (HIPV) that attract insect predators [8]. Lipoxygenases (LOXs) are enzymes that catalyze oxygenation of polyunsaturated fatty acids (PUFAs) such as linoleic acid [9] and are categorized as 9-LOX or 13-LOX depending on which carbon is being oxygenized. LOX-derived oxylipins play critical roles in plant growth, development, and plant defense against insect herbivores and pathogens, and are produced in response to a variety of biotic and abiotic stresses. The production of mycotoxins by fungi is partially regulated by oxylipins of both fungal and plant host origin [10–12], indicating a complicated interaction between hosts and pathogen using the same enzymes in both organisms.

In plants, LOX-mediated peroxidation of PUFAs results in production of a fatty acid hydroperoxide, which is further converted by one of several multigene enzyme families to produce a large number of diverse signaling molecules or molecules possessing direct antimicrobial and insecticidal activities [9]. The best studied branches of the LOX pathway are those leading to the production of jasmonic acid (JA) and green leaf volatiles (GLV), which are known to help plants defend against abiotic and biotic stresses, including fungi [13]. Jasmonic acid is a phytohormone involved in growth and development and regulates several defense pathways activated in plants in response to attack by pests and pathogens as reviewed in [14]. The synthesis of JA is initiated when α-linolenic acid (C18:3) is released from plastid membrane lipids by the action of phospholipase A1 (DAD1) and converted to 12-oxo-phytodienoic acid (12-OPDA) by LOX, allene oxide synthase (AOS) and allene oxide cyclase (AOC) [15]. The cumulative effects of genes from a genome-wide association analysis linked allelic variation in the jasmonic acid biosynthesis pathway with aflatoxin resistance in maize [16].

In addition to JA and GLVs, several other poorly studied oxylipins are postulated to regulate interactions between host plant and mycotoxin-producing pathogens as reviewed in [10]. The role of each specific LOX isoform is still being clarified. The deletion of LOX enzymes in maize has been found to influence plant development or pest /pathogen resistance [11, 13, 17–19]. In maize, a study of *ZmLOX3*, a LOX enzyme that belongs to the 9-LOX group, showed that disruption of this gene via a *Mutator* transposon insertion into the coding sequence of the gene resulted in drastic reduction of fumonisin production on kernels infected by *Fusarium verticillioides*. The *LOX3* disrupted maize line, *lox3-4*, in which the gene *ZmLOX3* was knocked out, was more resistant to southern corn leaf blight, anthracnose stalk rot and leaf blight, fumonisin contamination [17] and root rot pathogen, [19] but was more susceptible to *A*. *flavus* and aflatoxin production than the wild type (WT) maize control [17]. Jasmonic acid deficiency in an *opr7 opr8* double mutant as well as in *lox12* mutant resulted in complete loss of immunity or reduced resistance to another mycotoxin producing corn pathogen, *Fusarium verticillioides*, respectively [13] suggesting that JA is required for resistance against this seed and stalk pathogen. In general, however, studies characterizing each LOX gene in maize and its effect on biotic or abiotic stresses are still ongoing.

Linkage and association mapping are two complementary ways of testing the magnitude of the effect of a gene on the overall phenotypic expression of a trait. Linkage or quantitative trait loci (QTL) mapping accurately measures the effect of a larger genomic region on the trait of interest because the mapping population has a balanced proportion of alleles at all polymorphic loci. This provides stronger statistical power when compared to association mapping, but establishes much larger linkage blocks, due to relatively few generations of meiosis and thus recombination. In addition, QTL mapping only tests two alleles per locus in each mapping experiment. Association mapping utilizes all the diversity of many lines to identify multiple sequence polymorphisms and measure the phenotypic effect of favorable alleles associated with the phenotype. Due to a very large number of historical recombination events in an association panel, resolution can be within hundreds to several thousand base pairs.

Because of the importance of the LOX gene family in defense against pathogens, the objectives of this study were to characterize all genes that belong to the LOX gene family in maize through expression pattern and sequence polymorphisms and to map the phenotypic effect of these genes in up to four known QTL mapping populations and one association mapping panel. One gene, *ZmLOX10*, was sequenced in resistant and susceptible maize inbred lines, as mapping results indicated that this could be an important gene for fungal resistance.

## Materials and methods

### Database search for maize LOX genes and information

A search was carried out on five databases [20–24] to find any previously published LOX genes in maize and to seek out any gene or protein with LOX activity (GO annotation identification number 0016165) that could be responsible for the end product of any of the seven lipoxygenase pathway branches as reviewed by [10]. A literature search was also conducted to find any maize LOX genes that were not included in the online resources. A total of 13 ZmLOX genes were compiled, and the genes with their associated gene and protein identifiers and nucleotide positions on the B73 reference genome (AGPv3 and 4) can be found Table 1. Names of the genes are used in accordance with previous studies [13, 18, 23].

**Table 1:** A summary of the maize lipoxygenase gene family characterized in the current study.

Name	Gramene ID	Genebank accession	UniProt ID	Bin	Chr	Position^a^ from	Position^a^ to
**ZmLOX1**	Zm00001d042541 GRMZM2G156861	DQ335760	Q9LKL4	3.06	3	171,421,717 168,738,873	171,425,401 168,742,524
**ZmLOX2**	Zm00001d042540 GRMZM2G156861	DQ335761	A1XCH8	3.06	3	171,277,704 168.695,543	171,281,453 168,699,133
**ZmLOX3**	Zm00001d033623 GRMZM2G109130	AF329371	-	1.09	1	269,047,817 264,266,381	269,052,874 264,271,190
**ZmLOX4**	Zm00001d033624 GRMZM2G109056	DQ335762	M1HFG0	1.09	1	269,056,560 264,275,083	269,072,120 264,291,510
**ZmLOX5**	Zm00001d013493 GRMZM2G102760	DQ335763	A1XCI0	5	5	12,701,180 12,285,656	12,706,156 12,290,564
**ZmLOX6**	Zm00001d002000 GRMZM2G040095	DQ335764	A1XCI1	2.02	2	4,150,293 4,192,152	4,154,404 4,196,263
**ZmLOX7**	Zm00001d025524 GRMZM2G070092	DQ335765	A1XCI2	10.04	10	121,268,606 120,237,308	121,272,616 120,241,527
**ZmLOX8**	Zm00001d003533 GRMZM2G104843	DQ335766	A1XCI3	2.04	2	47,105,187 45,820,737	47,109,372 45,825,105
**ZmLOX9**	Zm00001d027893 GRMZM2G017616	DQ335767	A1XCI4	1.02	1	16,948,608 16,573,827	16,955,122 16,580,722
**ZmLOX10**	Zm00001d053675 GRMZM2G015419	DQ335768	A1XCI5	4.09	4	238,805,319 233,626,682	238,809,230 233,629,283
**ZmLOX11**	Zm00001d015852 GRMZM2G009479	DQ335769	Q06XS2	5.04	5	126,372,618 123,239,668	126,376,084 123,243,697
**ZmLOX12**	Zm00001d041204 GRMZM2G106748	DQ335770	A1XCI7	3.04	3	105,356,377 93,841,905	105,359,732 93,845,764
**ZmLOX13**	Zm00001d031449 GRMZM5G822593	-	-		1	190,316,537 188,148,388	190,321,767 188,153,483

Column headers include Gramene ID, (Maize B73 reference sequence versions 4, listed first and 3) and UniProt protein ID (when available); these are used as unique identifiers of each lipoxygense gene identified in the study. Bin location indicates genetic mapping location according to MaizeGDB, and position (from and to) indicates the physical interval in relation to the B73 maize reference genome.

^a^Physical position in base pairs according to the B73 reference sequence (version 4, top, and version 3, bottom).

Four ZmLOX genes (*ZmLOX3*, GRMZM2G109130; *ZmLOX4*, GRMZM2G109056; *ZmLOX9*, GRMZM2G017616; and *ZmLOX13*, GRMZM5G822593, which was called LOX2 in the previous B73 reference sequence annotation) are found on chromosome 1. *ZmLOX3* and *4* are only 3.7 kb apart; at such close proximity, neither QTL nor association mapping has a good possibility to distinguish the genetic effects of the two. Genes *ZmLOX6* and *8* (GRMZM2G040095 and GRMZM2G104843) are both located on chromosome 2. *ZmLOX1* (GRMZM2G156861 or ZM00001d042541); *ZmLOX2* (GRMZM2G156861 or ZM00001d042540) and *ZmLOX12* (GRMZM2G106748) are all found on chromosome 3. *ZmLOX1* and *2* are ~140 kb apart, and thus the QTL mapping analysis will be unable to separate the effects; association mapping should, however. There is one gene on chromosome 4 (*ZmLOX10*, GRMZM2G015419). *ZmLOX5* and *ZmLOX11* (GRMZM2G102760 and GRMZM2G009479, respectively) are both located on chromosome 5. *ZmLOX7* (GRMZM2G070092) is located on chromosome 10. All this information, summarized in Table 1, was used to identify the coordinates of these genes in the maize B73 reference genome and extract the reference DNA sequence of each gene for BLAST alignment and polymorphism identification.

Sequence alignment was carried out for all genes on the same chromosome with coordinates that were physically close to each other to ensure they were not the same gene given different names by different authors and databases. LOX genes with high sequence homology included *ZmLOX4* and *5* on chromosome 1 and 5, respectively, and *ZmLOX1* and *2*, a pair of closely linked genes on chromosome 3 (~140kb apart). Aligning the sequences of these genes against each other was also done to explore the possibility that one arose from the other in a recent duplication event. In the most recent version of the B73 maize reference sequence (AGPv 4), *ZmLOX1* and *2* have been given unique gene identifiers. Before this, they were mistakenly considered to be the same gene (GRMZM2G156861) with a very large intron (>100kb). The sequence separating *ZmLOX1* and *2* and the large (~11 kb) intron present in *ZmLOX4* between exon 2 and 3 of the canonical transcript reported in the V4 of the B73 maize reference sequence, or exons 1 and 2 of several of the other reported transcripts of *ZmLOX4*, were subjected to a BLAST search to determine if the introns are due to the presence of a transposable element in the maize genome.

**Fig 1:**
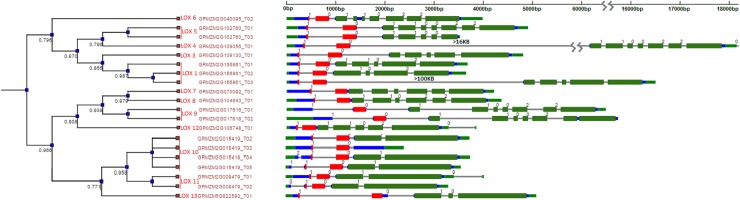
Phylogenetic tree analysis of all 13 maize LOX genes, including gene and transcript I.D. for each of the gene. The tree was constructed using the PIECE (Plant Intron and Exon Comparison Evolution) database. Green/blue color are 5’ and 3’ untranslated regions (UTRs), gray color corresponds to gene introns, and purple color to gene exons.

In order to gain more insight into relationships between genes and possible gene functions, two more databases were used in the characterization of the LOX gene family. To determine gene expression patterns in different tissues and developmental stages, the genome wide atlas of LOX transcription during maize development adapted from [25, 26] was searched for expression patterns of each LOX gene identified in maize. Finally, the PIECE (Plant Intron Exon Comparison and Evolution) Database [27] http://wheat.pw.usda.gov/piece was used to construct a phylogenetic tree (Fig 1) to determine structural relationships between the LOX genes and to provide clues about the evolutionary history of the genes. The PIECE database uses the pfam database (V26.0) [28] to classify all the plant genes and the FastTree program [29] to build the phylogenies.

### Genetic linkage mapping

Phenotypic and genotypic data previously published from the four F_2:3_ QTL mapping populations were combined with new genetic data for each ZmLOX sequence. Single nucleotide polymorphisms (SNP) from the candidate gene association analysis were converted to individual SNP assays using the KASP system from LGC Genomics (Hurts, UK). These use fluorescent labels on two unique forward primers binding to each allele of the SNP pair, and one common unlabelled reverse primer. The KASP assays were used to screen the parents of four QTL mapping populations, and polymorphisms detected between the parents were then scored in the corresponding F_2:3_ QTL mapping families. In addition, insertion/deletion (InDel) markers from genes *ZmLOX1*, *2*, *3* and *4*, and short sequence repeat (SSR) markers within 1,000kb upstream and downstream of the coordinates for each of the candidate genes were sought in MaizeGDB [25] and used for linkage mapping analysis when no polymorphic SNPs or InDels were found for a given mapping population. Due to the resolution of linkage mapping, gene pairs separated by less than 1000 kb and that appeared to be duplications, which included gene pairs *ZmLOX1* and *2* and *ZmLOX3* and *4*, were treated as a single QTL, as it would not be possible to tell which of the genes is responsible for the phenotypic effect on the trait (if either) using linkage mapping.

The four QTL mapping populations included MpB, a cross between Mp313E (resistant to aflatoxin contamination) x B73 (susceptible) [30]; MpV, Mp313E (resistant) x Va35 (susceptible) [31]; MpT, Mp715 (resistant) x T173 (susceptible) [32]; and MpNC, Mp717 (resistant) x NC300 (susceptible) [33]. All markers were amplified via PCR according to the manufacturers’ suggestions **(**Integrated DNA Technologies, Inc. Coralville, Iowa, USA). The PCR products of the SSR and InDel markers were electrophoresed and visualized on a 4% agarose gel with ethidium bromide. SNP markers were visualized using the OMEGA plate reader by BMG Labtech GMBH, (Orthenberg, Germany). The allele information obtained for every individual in the mapping populations in which the markers segregated was used to map and test the phenotypic effects on aflatoxin resistance for each candidate gene. Markers used to test each ZmLOX gene, including type, location, and in which mapping population they were run, are found in Table 2. Quantitative trait analysis for each of the ZmLOXs was carried out using the QTL cartographer V2.5 (Statistical Genetics, NCSU, Raleigh, NC, USA) using composite interval mapping to estimate the 0.05 significant threshold for each QTL.

**Table 2:** List of SNPs, InDels, and SSRs used to map the phenotypic effect of each ZmLOXs.

Gene Name	Marker	Bin	Segregating population	Est Marker position^a^	LOD Score^b^, Interval^c^, and R^2^^d^
**ZmLOX1**	InDel FxLOX_2738, SSR bnlg1063	3.06	MpB, MpT	168,862,738 174,813,098	MpB: 3.5 LOD, 57 Mbp, 3.4%. MpT: 5.8 LOD, 30Mbp, 13.6%
**ZmLOX2**	InDel FxLOX_2738, SSR bnlg1063	3.06	MpB, MpT	168,862,738 174,813,098	MpB: 3.5 LOD, 57 Mbp, 3.4%. MpT: 5.8 LOD, 30 Mbp, 13.6%
**ZmLOX3**	SSR phi037, SNP PHM4926_16	1.08	MpT (NS), MpVa (NS)	226,891,043 237,799,545	
**ZmLOX4**	SSR phi037, SSR glb1_2	1.08	MpT (NS), MpVa (NS)	226,891,043 257,540,930	
**ZmLOX5**	SNP PZB00094_1. SNP PZA01438_1	5.00	MpT, MpB (NS)	7,512,816 2,209,546	MpT: 2.4 LOD, 10 Mbp, 10.6%
**ZmLOX6**	SSR phi402893	2.00	MpB (NS)	1,350,406	
**ZmLOX7**	SSR umc1453	10.04	MpT (NS), MpNC (NS)	115,571,589	
**ZmLOX8**	SSR bnlg1018, SSR umc1783, SSR bnlg1909. SSR umc2247	2.02	MpB, MpNC (NS), MpT, MpVa (NS)	40,890,003 33,534,101 47,170,490 29,524,017	MpB: 9.0 LOD, 25 Mbp, 8.5%. MpT: 2.7 LOD, 15 Mbp, 1.6%
**ZmLOX9**	SSR umc1976	1.00	MpT (NS), MpNC (NS), MpVa	21,419,759	MpVa: 3.8 LOD, 15 Mbp, 5.8%
**ZmLOX10**	SSR umc2287	4.09	MpB, MpT, MpVa (NS)	213,902,712	MpB: 7.8 LOD, 30 Mbp, 16.3%. MpT: 2.4 LOD, 20 Mbp, 5.7%
**ZmLOX11**	SNP PZA02164_16	5.04	MpB (NS)	127,291,673	
**ZmLOX12**	SSR umc1968	3.03	MpB, MpNC (NS)	95,266,767	MpT: 3.6 LOD, 100 Mbp, 6.6%
**ZmLOX13**	SSR bnlg1057	1.06	MpB (NS)	188,960,253	

Gramene ID, the Maize B73 reference sequence versions 4, listed first, and 3, listed second for each ZmLox are shown. Each gene mapped in up to four QTL mapping populations, the estimated marker starting positions, populations in which they segregate, and approximate physical interval spanned by the QTL on each chromosome.

^a^ Position based on the maize V3 reference sequence.

^b^LOD score was set at a default threshold (2.5) of the composite interval mapping (CIM).

^c^Interval is the estimated physical distance the QTL spans; larger distances makes it much less likely that any single gene in the interval is the causal gene.

^d^ R^2^ value indicates the percentage of phenotypic variation explained by the QTL at the peak LOD for the trait in the mapping family. Note: this is the value associated with the peak score, and QTL with wide intervals are less likely to have the Lox gene directly at the peak and thus the R^2^ associated with the Lox gene may be lower.

### Aflatoxin association mapping

An in-house maize HapMap database was created to store Genotype By Sequencing (GBS) data for 287 maize inbred lines that form the aflatoxin association mapping panel described in [7]. It was used to identify SNPs or Insertion/Deletion (InDel) polymorphisms within the coordinates of each of the LOX genes (and extending up to 100 kb up-and downstream if insufficient polymorphism were found within the gene). An average of 56 polymorphisms was found for each candidate gene, and the allelic variant for each one in the 273 maize inbred lines was extracted. Only SNPs with a minor allele frequency of greater than 5% were used for association mapping. The panel of 273 diverse inbred lines had been testcrossed to a common tester and phenotyped for aflatoxin levels in inoculated, replicated field trials and reported in [7]. The TASSEL software package [34] was used for aflatoxin association mapping for each of the candidate genes, using the general linear model (GLM) and mixed linear model (MLM) as described in [35].

### Transcription profiling using RNAseq

Ears from line Mp719 (resistant) and Va35 (susceptible) were inoculated with *A*. *flavus* strain NRRL 3357 (toxigenic), *A*. *flavus* strain NRRL 21882 (nontoxigenic), or water 18 days after pollination (DAP). Inoculated kernels from 3 biological replicates were collected 3 days after inoculation (DAI). Kernels were flash-frozen in liquid nitrogen and total RNA was extracted from 100 mg of ground tissue using the Arum^TM^ Total RNA Fatty and Fibrous Tissue Kit (Bio-Rad Laboratories, Inc., Hercules, CA, USA), following the manufacturer’s protocol. Library construction was performed using the Illumina TruSeq Stranded prep kit (Illumina, Inc., San Diego, CA, USA) and 100 bp single-end sequencing was performed on the Illumina Hi-Seq 2000 with associated chemistries. Reads from all samples were aligned to the B73 reference genome (Zea_mays.AGPv3.23; http://plants.ensembl.org/Zea_mays/Info/Index) using TopHat 2 (v2.0.13), [36] and read counts were obtained using HT-Seq (v0.6.1), [37]. Differential expression of genes was determined using JMP Genomics software (v7.1, SAS Institute Inc., Cary, NC USA) with the Basic RNA-seq workflow. A mixed model was used for Analysis of Variance with the fixed effects being genotype, treatment and inoculation and the random effects being replicates. Data was modeled with a Negative Binomial distribution and genes were considered differentially expressed if they met a threshold of p-value < 0.0001 and a log2 FC ≥ ±1.

### Sequencing of *ZmLOX10*

The sequence of the *ZmLOX10* gene was obtained from five maize genotypes (two resistant genotypes, Mp313E and Mp715, and three susceptible, B73 Va35 and T173, to *A*. *flavus* infection and aflatoxin accumulation) following cloning of PCR products amplified from within exons of *ZmLOX10*. Primers for PCR were designed based on the B73 reference sequence and extensive BLAST alignment used to ensure that the sequence of this gene, and not homologous family members, were being amplified (S1 Table). Amplicons of up to 3 Kb were run on agarose gels, single bands of expected size were excised and cleaned using the QIAquick Gel Extraction Kit (Qiagen, Valencia, CA, USA). Amplicons were ligated into pGEM-T Easy, transformed into *E*. *coli*, and colonies grown on LB-Amp plates at 37°C for 12–16 hours. Four to eight colonies were transferred to LB-Amp broth and cultured individually at 37°C for an additional 16 hours. Recombinant plasmid DNA from colonies was isolated and purified with a QIAprep Spin Miniprep Kit (Qiagen, Valencia, CA, USA), quantified by UV spectroscopy, and sent to a service provider for dideoxy terminator sequencing. Sequences were aligned using EditSeq and MegAlign and assembled with SeqMan of the DNAStar sequencing package (Madison, WI, USA).

## Results

### Identification of maize lipoxygenase genes

The gene structure for each ZmLOX, which included the number of introns and exons present within each gene sequence, was used to create a phylogenetic relationship tree (Fig 1). The Pfam database [28] used by the PIECE software [27] to create dendrograms often enters multiple transcripts of each gene separately into the phylogenetic tree (as can be seen Fig 1) which then appear as clusters of highly related entries. It must be noted, however, that some of the transcripts may be based on splice sites predicted by computer algorithm and not verified by the presence of a sequenced protein or active transcript. Clustering occurred based on LOX function, as genes from the 9-LOX and 13-LOX functional groups separated into two distinct clusters.

### Mapping

**Fig 2:**
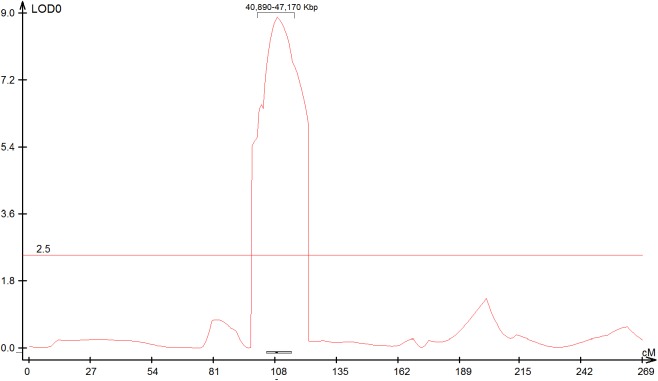
Composite interval mapping results of the MpB population (chromosome 2) for natural log transformed aflatoxin level values in one environment. The x axis represents the genetic length of the chromosome and the y axis represents the LOD. The horizontal line indicates LOD significance threshold of 2.4. GRMZM2G104843 in bin 2.04 mapped right under a QTL spanning 6.2 mbp.

Linkage mapping was used to determine the phenotypic effect of each LOX gene and to confirm the QTL position in the maize genome. Mapping results of the SNP, InDel and SSR markers identified within or linked to each gene sequence in one or more mapping populations are presented in Table 2. Two SSRs linked to *ZmLOX8*, also known as the mutant *tasselseed1* (*ts1*) in bin 2.02 [38], mapped to one of the previously identified QTL in chromosome 2 of the MpB population with a LOD score of 9.0 (Fig 2), and explained 17.7% of the phenotypic variation observed in this population in one environment (Table 2), and identified lower but still significant QTL in 5 other environments or averages over environments. The QTL was associated with an additive gene action and the allele associated with a reduction in aflatoxin levels came from the resistant parent Mp313E. This gene was also found under a QTL in MpT population with a LOD score of 2.7. The QTL interval in MpB spanned 20 cM, or about ~ 6 million base pairs (Fig 2) and is narrowly delineated for a mapping population of this size, but is still large enough to hold many genes, any one of which could be the cause of the QTL. *ZmLOX8* is a very possible candidate to be the causal gene of the QTL, however, as it falls directly under the peak of the QTL, and also from its known function. *ZmLOX8* is part of the pathway that provides substrates for the synthesis of JA [17], which is the major phytohormone directly involved in plant resistance to necrotrophic fungi and chewing insects [13].

An SSR linked to *ZmLOX10* in bin 4.09 mapped under a QTL of LOD 7.8 that explained 15.5% of the phenotypic variation in MpB, and another QTL in population MpT with a LOD of 2.4 (Table 2). The resistance came from the resistant parents in both cases. The QTL interval was even wider than that of *ZmLOX8*, and large enough to contain many candidate genes, but *ZmLOX10* is a possible causal gene. LOX10 is the only maize LOX isoform responsible for the biosynthesis of green leaf volatiles (GLVs) [18], a group of compounds that possess both anti-insect and anti-fungal properties [39]. These GLVs have been reported as signals to induce expression of other defensive genes as well [40].

An InDel and an SSR linked to *ZmLOX1*and *2* was found beneath a QTL in bin 3.06 in population MpT with a LOD of 5.8 and another in population MpB with a LOD of 3.5 (Table 2). These explained 13.6% and less than 5% of the phenotypic variation, respectively (Table 2). *ZmLOX1* and *2* are both in the predominantly 9-LOX group that have dual region-specificity (e.g. can catalyze oxygenation at both 9- and 13-C position of fatty acids) that responds to biotic stresses, wounding, JA, and ABA [41]. The QTL interval is very wide and contains both genes, but also potentially many other genes.

Three other previously published QTL were highlighted in this linkage mapping exercise after markers linked to genes in bin 5.00 (*ZmLOX5*), bin 1.00 (*ZmLOX9*), and bin 3.03 (*ZmLOX12*) were found to map within the interval of these QTL with LOD scores ranging from 2.4 to 3.8 in one mapping population each. *ZmLOX5* and *9* were found in particularly narrow QTL intervals. Several of the markers linked to all other ZmLOX genes were also polymorphic in the four mapping populations and mapped to the correct locations, but no QTL were identified (Table 2).

For the association mapping, a total of 726 SNPs were identified within the genetic sequence of all the ZmLOX genes using the in-house HapMap database (S2 Table). Of the 726 SNPs, 28 were identified as associated to aflatoxin accumulation resistance according to the general linear model (GLM) of TASSEL, with p-values that ranged between 1.26 x 10^−4^ ≤ p ≤ 9.71 x 10^−4^ (Table 3). These fell within 100,000 base pairs of nine ZmLOX genes (*1/2*, *3/4*, *5*, *6*, *7*, *8*, *9*, *10* and *13*); these include five of the six genes that fell within a QTL as well (*ZmLOX1/2*, *5*, *8*, *9* and *10*) and this may serve to increase the confidence in the two mapping methods. An insufficient number of SNPs were extracted from within or near the sequences of *ZmLOX3/4*, *11* and *12*; accordingly, *ZmLOX11* and *12* were the only two genes with no SNPs associated with reduced aflatoxin, and only one was associated from *ZmLOX3/4* (Table 3). The association probabilities from all genes were not sufficiently low to be used as indicative of anything more than a trend. In addition, only ~5% or less of the SNPs extracted from every gene with more than 20 SNPs tested were associated, with the exception of *ZmLOX8*, for which just over 8% were associated.

**Table 3:** Summary of gene association analysis for the 13 ZmLOX genes studied.

Gene	# SNPs extracted	% associated	Trait	Marker^a^	Chr	Marker p	Marker R^2^
ZmLOX1/2	37	5.4%	Star09LSM	S3_168962439	3	3.80E-04	0.039
			AveLSM	S3_168923153	3	6.69E-04	0.033
ZmLOX3/4	20	5.0%	Lubb10LSM	S1_264183077	1	4.85E-04	0.041
ZmLOX5	89	3.3%	Lubb10LSM	S5_12219354	5	5.92E-04	0.046
			Star09LSM	S5_12192206	5	9.00E-04	0.032
			AveLSM	S5_12388000	5	9.29E-04	0.03
ZmLOX6	155	4.5%	Star09LSM	S2_4294282	2	5.33E-04	0.036
			AveLSM	S2_4211008	2	5.58E-04	0.032
			Lubb10LSM	S2_4187476	2	8.24E-04	0.042
			Lubb10LSM	S2_4292110	2	8.38E-04	0.043
			Lubb10LSM	S2_4291888	2	8.82E-04	0.042
			AveLSM	S2_4294282	2	8.86E-04	0.031
			Lubb10LSM	S2_4262909	2	8.90E-04	0.045
ZmLOX7	52	5.7%	CSta09LSM	S10_120219787	10	1.26E-04	0.053
			CSta10LSM	S10_120220589	10	4.39E-04	0.042
			AveLSM	S10_120219787	10	7.51E-04	0.027
ZmLOX8	37	8.1%	CSta09LSM	S2_45767527	2	3.43E-04	0.045
			AveLSM	S2_45780048	2	7.69E-04	0.032
			AveLSM	S2_45780061	2	7.98E-04	0.032
ZmLOX9	47	2.1%	Lubb09LSM	S1_16378473	1	7.78E-04	0.037
ZmLOX10	97	5.1%	AveLSM	S4_233636604	4	5.04E-04	0.033
			Star09LSM	S4_233636034	4	5.46E-04	0.037
			AveLSM	S4_233636686	4	5.52E-04	0.032
			Star09LSM	S4_233636078	4	7.57E-04	0.036
			AveLSM	S4_233636692	4	8.80E-04	0.03
			AveLSM	S4_233636547	4	9.71E-04	0.03
ZmLOX11	11	0.0%					
ZmLOX12	20	0.0%					
ZmLOX13	161	1.2%	AveLSM	S1_188114572	1	4.22E-04	0.033
			AveLSM	S1_188181812	1	4.30E-04	0.034

Number of SNPs extracted from a HapMap database of GBS data presented in Warburton et al., 2015, and the percentage of these that were associated at the p<10^−4^ are shown in the first two columns.

^a^Markers are named using position in the V2 maize reference sequence. Values for p and R^2^ were estimated by the general linear model and candidate gene association analysis, where each candidate gene was analyzed one at a time to avoid large experiment-wide false discovery errors.

### Expression

To gain further insights into the potential role of any LOX gene in maize interactions with *A*. *flavus*, we have mined the results of RNAseq transcriptome analyses of maize kernels infected by either toxigenic or atoxigenic strains of *A*. *flavus*, strain 3357 and strain 21881, respectively, or mock-treated (inoculated with water) under field conditions 3 days post infection (Table 4). The resistant inbred, Mp719, accumulated low levels of aflatoxin, while the Va35 susceptible line displayed a much higher level of the toxin (data not shown). The RNAseq data indicated that *ZmLOX1/2*, *6*, and *10* were downregulated in Mp719 inoculated with *A*. *flavus* (one or both strains) compared to the mock control, but not compared to Va35 inoculated with toxigenic *A*. *flavus*. These results point to a potential role of these genes in producing oxylipins that facilitate the pathogenesis processes or positively regulate aflatoxin biosynthesis as reviewed in [15, 42]. *ZmLOX7* was upregulated in Mp719 inoculated with the atoxigenic *A*. *flavus* only compared to water. *ZmLOX4*, *8* and *13* were also upregulated in Mp719 inoculated with one or both *A*. *flavus* strains compared to water, and also compared to Va35 inoculated with toxigenic *A*. *flavus*. These results suggest a 9-oxylipin producing 9-LOX, *ZmLOX4*, and 13-LOXs including putative JA producing isoforms *ZmLOX7*, *8* and *13*, may have a direct role in resistance to aflatoxin accumulation in maize.

**Table 4:** Expression data of ZmLOX genes.

		Expression levels in uninoculated maize. Data from MaizeGDB gene expression atlas (Stelpflug et al., 2015)							Expression level differences (Tmt 1 –Tmt2)			p value of the differences, *p< 10^−5^, ** p< 10^−6^		
Gene Name	chr.	Anthers	Dev. ear	Endosp. 25 DAP	Ovule	Silk	Tassel	Pollen	Mp719 I1—Mp719 H_2_0	Mp719 I2 -Mp719 H_2_0	Mp719 I2 -Va35 I2	Mp719 I1—Mp719 H_2_0	Mp719 I2 -Mp719 H_2_0	Mp719 I2 -Va35 I2
ZmLOX1	3	2.5	51.6	13.6	242.1	39.5	15.2	0.01	-1.87	-4.22	-1.91	ns	**	ns
ZmLOX2	3	2.5	51.6	13.6	242.1	39.5	15.2	0.01	-1.87	-4.22	-1.91	ns	**	ns
ZmLOX3	1	14.2	14.9	9.9	37.2	5.8	57.4	0.03	ns	ns	ns	ns	ns	ns
ZmLOX4	1	10.3	32.4	2.1	54.9	19.9	21.3	0.39	2.69	3.06	3.37	**	**	**
ZmLOX5	5	25.5	194.2	5.3	549.3	557.5	180.1	0.06	ns	ns	ns	ns	ns	ns
ZmLOX6	2	0.5	85.5	13.3	197.3	26.0	113.6	0	-1.64	-2.12	-1.61	ns	*	ns
ZmLOX7	10	6.9	0.4	0.9	0.7	0.4	1.6	0	2.76	2.21	2.33	*	ns	ns
ZmLOX8	2	4.3	3.0	2.2	2.0	3.1	13.6	0	2.42	2.26	3.98	**	*	**
ZmLOX9	1	9.5	16.4	3.1	6.2	18.9	3.2	0	ns	ns	ns	ns	ns	ns
ZmLOX10	5	0.3	33.1	6.4	132.1	669.1	53.4	0	-1.45	-2.29	-1.60	ns	*	ns
ZmLOX11	3	1.5	0.3	22.4	8.1	0.2	2.3	0.51	ns	ns	ns	ns	ns	ns
ZmLOX12	1	0.7	0.1	0.1	0.2	0	1.8	0.04	ns	ns	ns	ns	ns	ns
ZmLOX13	1	-	-	-	-	-	-	-	3.649	4.610	4.674	ns	*	*

Expression data of ZmLOX genes in different uninoculated tissues at different stages in the life of the maize plant from the MaizeGDB maize gene expression atlas (first seven columns following Gene Name and chromosome); and from RNAseq data from this experiment (last 6 columns).

Tissue and stages include mature anthers, developing ear (Dev. ear), endosperm 25 days after pollination (Endosp. 25 DAP), mature ovule, silk, post-meiotic tassel, and pollen. Treatments (abbreviated Tmt) for RNAseq data include Mp715 (resistant maize inbred line) inoculated with *A*. *flavus* strain 21882 (atoxigenic, I1); *A*. *flavus* strain 3357 (toxigenic, I2); or water; and Va35 (susceptible maize inbred line) inoculated with *A*. *flavus* strain 3357 (toxigenic, I2); or water. Expression level values and green color intensity (where darker green indicates higher expression levels) are both used to visualize the data.

*ZmLOX3*, *5*, *9*, *11*, and *12* were not significantly differentially expressed in any of the treatment comparisons. It was surprising to see no induction of the *ZmLOX3* gene previously shown by mutant analysis to be required for defense against *A*. *flavus* and aflatoxin accumulation [19]. However, because the RNAseq experiment included a single time point, it cannot be ruled out that some LOX genes are also responsive to infection with *A*. *flavus* at other times. To establish either positive or negative role of any of the LOX genes in maize resistance to aflatoxin contamination, continued field based screening of near isogenic knock-out mutants and wild types will be required.

### Sequence origins

Comparing the sequences of the 13 LOX genes found in maize allows us to speculate about the origin and evolution of these genes. This is an interesting family of maize genes, as they tend to come in highly conserved (>80% sequence similarity) pairs. This is common with tandemly duplicated genes in the maize genome, which may result in duplication following transposable element insertion (with or without subsequent excision). However, the LOX family seems to be an extreme example of this case, as nearly every ZmLOX gene has a highly similar duplicate, often very close on the chromosome.

Three ZmLOX genes are highly similar and may derive from each other. *ZmLOX3* is located in bin 1.09 and has 7 exons and 6 introns (all less than 1 kb). *ZmLOX4* is also located in bin 1.09 and falls 3.6 kb away from *ZmLOX3*. *ZmLOX5* is located in bin 5.00; it shares the highest homology with *ZmLOX4* of all ZmLOX pairs, with an amino acid sequence similarity of 94% [43]. Both genes *ZmLOX4* and *5* consist of 9 exons and 8 introns, but the second intron spans ~ 11 kb in *ZmLOX4* and only ~ 500bps in *ZmLOX5*. A BLAST search of this large intron from *ZmLOX4* was conducted on the GRAMENE database (a maize database) and the sequences from the maize transposable element database (TEDB,) [44]. It was found to match large introns of hundreds of genes spread throughout the maize genome and to several known transposable elements (TEs), in particular, the long terminal repeat (LTR) *gypsy*-like (RLG), and an unknown (RLX) transposable element. *ZmLOX3* is 80% similar to both *ZmLOX4* and *ZmLOX5*, excluding introns. The large intron in *ZmLOX4* with high sequence homology to the *gypsy*-like TE may be the cause of the duplication of *ZmLOX3* to form *ZmLOX4* via a short distance transposition event in which the sequence of the TE was left in the new *ZmLOX4*. There are no sequence similarities between the introns of *ZmLOX3* and the maize TEDB. There is a 123 bp sequence in the 5’ UTR of *ZmLOX5* with a 90% sequence homology to a class 2 TE. This may also indicate that a long distance transposition event created *ZmLOX5*, also probably starting from *ZmLOX3*. In contrast, *ZmLOX3*, *4* and *5* are only 40–67% identical to other ZmLOXs [45].

The linked genes *ZmLOX1* and *ZmLOX2* are the next most similar homologs in the ZmLOX gene family. They previously shared the same ID due to automatic prediction error and to their close physical proximity, but should now be regarded as separate genes, each with a complete gene structure and transcript set. The long sequence (136,130 bp) between the two linked genes in the two gene model was blasted against the maize TEDB and more than one hit was found with a Z value of 0.0 for sequences of between ~700 to over 14,000 base pairs. These included *gypsy*-like (RLG, especially the xilon-diguus, prem1, huck, grandem and cinful-zeon sequences) and *copia*-like (RLC, including ji and opie sequences) class one TEs; and *Mutator* (DTM), CACTA (DTC) and hAT (DTA) class two elements. It is probable that the sequence of an ancestral *ZmLOX1* or *2* gene has been disturbed by at least one TE insertion, creating the duplication event that led to two existing genes.

The gene pair *ZmLOX7* on chromosome 10 and *ZmLOX8* on chromosome 2 is a set of segmentally duplicated genes with near identical sequences, both having 7 exons and 6 introns (none longer than ~500 bp in the maize B73 reference genome). According to BLAST alignment, both genes including introns share 84% identity and excluding introns, they are 95% identical. Another set of duplicated genes are *ZmLOX10* on chromosome 4, with 3 exons and 2 introns, and *ZmLOX11* on chromosome 5, with 5 exons and 4 introns. These two genes share 94% of sequence identity. Similarities between these two pairs of genes were also reported by [46].

### Sequence of ZmLOX10

**Fig 3:**
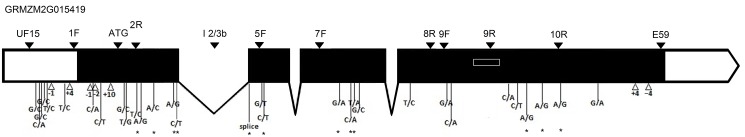
Sequence summary of the 5 maize inbred lines sequenced for *ZmLOX10*. Exons are filled black boxes, introns are black lines, and up-and down-stream untranslated regions are white boxes. Numbers above the gene indicate the primers used to amplify various amplicons spanning the gene (S1 Table), and the ATG start site is indicated. Triangles and numbers below the gene are insertions (or deletions, if negative), and SNPs are shown in their approximate locations with the two bases indicated. Asterisks below the gene indicate SNPs that cause a non-synonymous mutation, as numbered and described in Table 5. Small bar to the upper right of the figure indicates the length of 100 base pairs.

The sequence of the *ZmLOX10* gene, including 650 base pairs upstream and 235 base pairs downstream of the gene, was sequenced in its entirety from two resistant inbred maize lines and three susceptible maize inbred lines, including B73. The primers used to amplify fragments that were cloned and sequenced are shown in Fig 3 and S1 Table, and the entire sequence for five inbred lines shown in S3 Table (DNA and protein sequence). The sequence of the B73 line was identical to the reference sequence (V4) gene ID GRMZM2G015419, canonical transcript (T001) presented in the Gramene website (www.Gramene.org, release 52) (S3 Table). A summary of the sequence differences between the five inbred lines is presented in Fig 3 and Table 5.

**Table 5:** Summary of the single nucleotide polymorphisms (SNPs) found in the coding sequence of *ZmLOX10* in five inbred maize lines.

Non-syn. Mutation #	SNP ID	Base change	Consequence of mutation	B73	Mp313E	Mp715	T173	Va35	Amino acid substitution
1 2	PZE04239235711	T/G	synonymous	T	G	G	G	T	
2	PZE04239235714	G/C	synonymous	G	C	C	C	G	
3	S4_233625960	T/C	Missense	T	T	T	T	C	Serine to Proline
4	S4_233625974	A/G	synonymous	A	A	A	A	G	
5	S4_233623033	A/C	Missense	A	A	A	A	C	Histidine to Proline
6	PZE05239235918	C/G	synonymous	C	C	C	C	G	
7	PZE05239235988	A/G	Missense	A	G	A	A	G	Isoleucine to Valine
8	PZE04239235999	C/A	synonymous	C	A	C	C	A	
9	PZE04239236000	C/T	Missense	C	T	C	C	Y	Leucine to Phenylalanine
10	PZE04239236020	C/G	synonymous	C	G	C	C	G	
11	PZE04239236528	G/A	splice region/ synonymous	G	A	G	G	G	
12	S4_233626821	G/T	synonymous	G	T	G	G	G	
13	PZE04239236626	C/T	Missense	C	C	C	C	T	Proline to Leucine
14	PZE04239237135	G/A	Missense	G	G	G	G	A	Glutamate to Lysine
15	PZE04239237302	C/A	Missense	C	A	C	A	A	Aspartic Acid to Glutamate
16	PZE04239237307	T/A	Missense	T	A	T	A	A	Phenylalanine to Tyrosine
17	PZE04239237330	G/C	Missense	G	G	G	C	G	Alanine to Proline
18	PZE04239237605	T/C	synonymous	T	C	C	T	C	
19	PZ304239237914	G/A	synonymous	G	A	G	G	G	
20	PZE04239237947	C/A	synonymous	C	C	C	C	A	
21	PZE04239238199	C/A	synonymous						
22	PZ304239238217	C/T	synonymous	C	C	T	T	C	
23	PZE04239238278	A/G	Missense	A	A	G	G	G	Isoleucine to Valine
24	PZE04239238381	G/A	Missense	A	A	A	G	A	Arginine to Lysine
25	PZE04239238454	G/A	synonymous	G	A	G	A	G	
26	PZE04239238687	A/G	Missense	A	G	A	A	G	Glutamate to Glycine
27	PZE04239238835	G/A	synonymous	G	G	G	G	A	
AA Length				905	904	855	905	931	
Protein PI				6.11	6.11	6.11	6.11	6.24	
Mol. Weight				102,069.36	101,737.66	96,623.06	102,083.38	105,093.95	

The SNPs that lead to a non-synonymous (missense) difference in the final proteins are numbered in the first column and location shown in Fig 3. The SNP identification, change in base compared to the B73 reference sequence, and consequence of the mutation are shown, as well as the genotype of the SNP at that location in five inbred maize lines (B73, Mp313E, Mp715, T173, and Va35). Predicted amino acid change compared to the B73 predicted protein is also shown in the last column. In the last three rows, the overall length, isolelectric point (PI), and molecular weight of the predicted protein is shown.

Part of intron 1 in B73 and Va35 could not be sequenced; however, we know from electrophoresis that the length of these amplicons is the same as the other genotypes, so no insertion or deletion longer than ~ 3bp can be present in these two genotypes. Three replicated PCR reactions were each cloned four times and were sequenced from both ends, and thus, there were at least 8 and up to 24 aligned sequences for each amplicon in each inbred line. In addition, the cDNA from *ZmLOX10* was sequenced following isolation of RNA from mature leaves, for confirmation of splice variants. Mature leaves are known to express ZmLOX10 and not the highly homologous *ZmLOX11*, avoiding alignment errors.

There were a total of 27 SNP polymorphisms found between the sequences of the five inbred lines in the coding regions, and all had been reported in the maize hapmap variants [47]. Of these 27 sequence changes, there were 13 possible functional polymorphisms including one splice variant in Mp313E that has the possibility of changing transcript T001 to another one; and 812 missense polymorphisms caused by a non-synonymous mutation (S3 Table, Fig 3). The ninth SNP is in the splice junction between intron 1 and exon 2 in Mp313E and has the possibility of changing transcript T001 to another one; however, the size and sequence of the cDNA fragment made from the *ZmLOX10* RNA extracted from mature maize leaves and the sequence were no different in Mp313E than the other 4 genotypes (data not shown). The 12 missense mutations cause various amino acid substitutions, some of which may cause a change in the configuration of the enzyme (Table 5). There are six previously unknown insertions (ranging between 1 and 10 bp), and two previously unknown SNPs in exon 1, before the start ATG codon (Fig 3). Some of these DNA sequence changes may cause changes to the final protein.

## Discussion

All ZmLOXs were found on six of the ten chromosomes present in the maize genome, and all mapped in our mapping populations to these locations as expected. Maize LOX genes are divided into two major functional groups: 9-hydroperoxide-generating (9-LOX) and 13- hydroperoxide-generating (13-LOX), depending on the carbon where they catalyze molecular dioxygenation. *ZmLOX1*, *2*, *3*, *4* and *5* all belong to the predominantly 9-LOX group, whose function is still not well known, while *ZmLOX7*, *8*, *9*, *10*, *11*, and *13* all belongs to the 13-LOX group, which produce JA and GLVs [17, 38, 45, 46]. Compounds produced by the various LOX pathways belonging to the 13-LOX group include those derived from the hydroperoxide lyase (HPL) and allene oxide synthase (AOS) branches, which produce GLV and JA, respectively. These compounds play a very important role in plant immunity against predatory insects and fungi [48, 49]. Metabolic pathway analysis has also shown that the JA synthesis pathway was significantly associated with resistance to aflatoxin accumulation in maize [16]. Mapping results in the present study suggest that genes *ZmLOX1*, *2*, *5*, *8*, *9*, and *10* map within QTL and are also associated with a reduction in aflatoxin levels in a candidate gene association study. Mapping results are strongest for genes *ZmLOX8* and *10*, both of which are 13-LOXs.

Genes *ZmLOX8* and *ZmLOX10* are reported to have direct and indirect roles in plant defense against herbivory and fungal resistance by producing the substrates used in the biosynthesis of JA and GLVs, respectively [18]. There is evidence that *ZmLOX8* and *ZmLOX10* work synergistically, at least, in terms of wound-induced JA biosynthesis, although the genes are found on different chromosomes in the maize genome. A lack of expression of the GLV-producing *ZmLOX10* leads to diminished levels of wound-induced expression of *ZmLOX8*, a major JA producing enzyme [18]. Unfortunately, such an epistatic interaction could not be detected in the QTL mapping populations of the size used in this study. *ZmLOX8* mapped directly under a QTL of LOD value 9.0 (Table 3). Biochemical analyses of *lox10* knock-out mutants clearly showed that *ZmLOX10* is the only LOX enzyme isoform required for production of GLVs [18]. Interestingly, its closest segmentally duplicated homolog, *ZmLOX11*, is not involved in GLV biosynthesis as *lox10* mutants are completely devoid of GLVs despite normally functioning *ZmLOX11* [18, 47]. This may be because *ZmLOX11* is not expressed in mature maize leaves [26]. *ZmLOX10* was found beneath a QTL for aflatoxin accumulation resistance with a LOD value of 7.8; however, it was a large QTL interval and other genes may influence it as well. Past characterization studies of the ZmLOX genes did not include extensive sequencing of *ZmLOX10*, which is presented here from 5 maize inbred lines for the first time.

*ZmLOX5* belongs to the 9-LOX family and is expressed in silks, husk and tassel as shown in Fig 3 [26, 43] and mapped directly under another QTL found in bin 5.00 with a LOD value of 2.4. The *A*. *flavus* fungus is known to infect maize ears through the husk or to use the silk channel of developing kernels to gain entry into the ear [49], and thus reduced aflatoxin levels are expected to be associated with resistance factors expressed in silks and husks. The near identical homolog *ZmLOX4* was neither associated nor linked to a QTL for aflatoxin accumulation resistance, and it has a very different expression pattern than *ZmLOX5*, as it is expressed primarily in the roots [43]. This may explain the lack of association with aflatoxin levels in maize grain, and we may speculate that *ZmLOX4* may have more to do with resistance to insects that attack corn roots. *ZmLOX3* is expressed for the most part in the developing embryos, germinating seed and the innermost husk; no QTL for reduced aflatoxin in the ear was reported over this gene either. Similar gene pairs *ZmLOX7* and *8* showed alternate expression patterns, with *ZmLOX7* expressed at very low levels overall, but slightly higher in the embryo and anthers, and *ZmLOX8* expressed at high levels in older leaves. If *ZmLOX8* is the causal gene for the strong QTL found directly above it, the phenotypic effect may be indirect, possibly even via interaction with *ZmLOX10*. On the other hand, similar gene pairs *ZmLOX10* and *11* were both highly expressed in young leaves, but also in silks in the case of *ZmLOX11* and older leaves in the case of *ZmLOX10* [26, 45].

Although most of the ZmLOXs identified in this study had only modest or no effect on aflatoxin accumulation resistance, and explained between 0 and 5% of the phenotypic variation observed in the populations measured, it may still be informative to verify the effect of resistance alleles through the creation of transgenic lines, near isogenic lines (NILs) carrying specific alleles identified in this or other studies, or knock-out mutants (currently being generated) to verify the effect of one or more of these genes in a different background other than the background present in the mapping populations of this study. *ZmLOX8* may have a large enough effect and strong enough mapping evidence to justify further studies. Unfortunately, *lox8* knock-out mutants (*ts1* allele) [38] are devoid of JA in tassel tissues and are male-sterile, unable to produce pollen, and thus, making testing for aflatoxin contamination under field conditions difficult. While this study and preliminary testing of the LOX knock-out mutants suggest the involvement of specific ZmLOXs in the interactions with *A*. *flavus* colonization and production of aflatoxin [11 and unpublished], it is evident that the complicated functions of oxylipins, including their role in signaling cross-talk with the infecting *A*. *flavus*, indicate a need for additional field and laboratory based experiments to clarify how these genes can be utilized for gains in resistance to this fungus via marker assisted selection.

## Supporting information

S1 Table Sequence of the primers used to amplify *ZmLOX10*, GRMZM2G015419 from five maize inbred lines. These sequences were used for subsequent cloning and sequencing. Positions are indicated from the Maize B73 reference sequence version 3, and indicate the position along the gene as counted from 3000 upstream from the start codon.
(XLSX)

S2 Table All SNPs from within 100 Kb of the ZmLox genes characterized in this study. RS number, chromosome, position (according to B73 reference sequence 3) and allele calls for all lines used in the association study are shown.
(XLSX)

S3 Table DNA and protein sequence of *ZmLOX10*, GRMZM2G015419 from five maize inbred lines. Sequences were aligned using CLUSTAL multiple sequence alignment by Kalign (2.0). DNA sequence starts 650bp upstream of first EXON. ATG start codon highlighted in blue. TGA stop codon highlighted in yellow. Deletions are indicated with dashes–and unsequenced bases with gray highlighted dashes.
(DOCX)
