# Supplementary material for: Characterization of the maize lipoxygenase gene family in relation to aflatoxin accumulation resistance
Source: PLoS One. 2017 Jul 17;12(7):e0181265. doi: 10.1371/journal.pone.0181265 (PMC5513560; doi:10.1371/journal.pone.0181265)
Supplement: S3 Table — Sequences were aligned using CLUSTAL multiple sequence alignment by Kalign (2.0). DNA sequence starts 650bp upstream of first EXON. ATG start codon highlighted in blue. TGA stop codon highlighted in yellow. Deletions are indicated with dashes–and unsequenced bases with gray highlighted dashes. (DOCX) [file pone.0181265.s003.docx]

DNA sequence alignment of GRMZM2G015419, *ZmLox10*:

CLUSTAL multiple sequence alignment by Kalign (2.0); Reference sequence the B73 v3 reference; Coding sequence is the expected sequence of the coding bases only.

SNPs or InDels are highlighted in yellow; start codon highlighted in blue; dashes are insertions; gray highlighted dashes are missing sequence. Yellow highlighted SNPs are numbered as in Table 5.

Reference GGACATTGACCGACGCAGGCCAACAAAGACAGGACATGTTTTAGGATCGGACTAGACCAC

Coding ------------------------------------------------------------

B73 -----------------GGCCAACAAAGACAGGACATGTTTTAGGATCGGACTAGACCAC

Va35 -----------------GGCCAACAAAGACAGGACATGTTTTAGGATCGGACTAGACCAC

T173 -----------------GGCCAACAAAGACAGGACATGTTTTAGGATCGGACTAGACCAC

Mp715 -----------------GGCCAACAAAGACAGGACATGTTTTAGGATCGGACTAGACCAC

Mp313E -----------------GGCCAACAAAGACAGGACATGTTTTAGGATCGGACTAGACCAC

Reference TGTTTCTACTCTTTAGGCTCGCACGACACGACCTAAAATTTTTTAGACCTTCTTAGTCCA

Coding ------------------------------------------------------------

B73 TGTTTCTACTCTTTAGGCTCGCACGACACGACCTAAAATTTTTTAGACCTTCTTAGTCCA

Va35 TGTTTCTACTCTTTAGGCTCGCACGACACGACCTAAAATTTTTTAGACCTTCTTAGTCCA

T173 TGTTTCTACTCTTTAGGCTCGCACGACACGACCTAAAATTTTTTAGACCTTCTTAGTCCA

Mp715 TGTTTCTACTCTTTAGGCTCGCACGACACGACCTAAAATTTTTTAGACCTTCTTAGTCCA

Mp313E TGTTTCTACTCTTTAGGCTCGCACGACACGACCTAAAATTTTTTAGACCTTCTTAGTCCA

Reference AACCCGTTTGGAACGAACCACAAAGTGGACCCGGCCAATTCCCATCACTAGTCCAAACAC

Coding ------------------------------------------------------------

B73 AACCCGTTTGGAACGAACCACAAAGTGGACCCGGCCAATTCCCATCACTAGTCCAAACAC

Va35 AACCCGTTTGGAACGAACCACAAAGTGGACCCGGCCAATTCCCATCACTAGTCCAAACAC

T173 AACCCGTTTGGAACGAACCACAAAGTGGAYCCGGCCAATTCCCATAACTAGTCCAAACAC

Mp715 AACCCGTTTGGAACGAACCACAAAGTGGACCCGGCCAATTCCCATAACTAGTCCAAACAC

Mp313E AACCCGTTTGGAACGAAGCACAAAGTGGACCCGGCCAATTCCCATCACTACTCCAAACAC

Reference ACAATCCAGGACATACGACATACACTACACACACAGAGTCACAGACAGACCAGCGCTCTG

Coding ------------------------------------------------------------

B73 ACAATCCAGGACATACGACATACACTACACACACAGAGTCACAGACAGACCAGCGCTCTG

Va35 ACAATCCAGGACATACGACATACACTACACACACAGAGTCACAGACAGACCAGCGCTCTG

T173 ACAATCCAGGACATACGACATACACTACACACACAGAGTCACAGACAGACCAGCGCTCTG

Mp715 ACAATCCAGGACATACGACATACACTACACACACAGAGTCACAGACAGACCAGCGCTCTG

Mp313E ACAATCCAGGACACACGACATACACTACACACACAGAGTCACAGACAGACCAGCGCTCTG

Reference CATGGCCATCATTCTGGTGACCACGCAAAAAGGACCAGCCATTCCCGGGCAAAGATGGTT

Coding ------------------------------------------------------------

B73 CATGGCCATCATTCTGGTGACCACGCAAAAAGGACCAGCCATTCCCGGGCAAAGATGGTT

Va35 CATGGCCATCATTCTGGTGACCACGCAAAAAGGACCAGCCATTCCCGGGCAAAGATGGTT

T173 CATGGCCATCATTCTGGTGACCACGCAAAAAGGACCAGCCATTCCCGGGCAAAGATGGYT

Mp715 CATGGCCATCATTCTGGTGACCACGCAAAAAGGACCAGCCATTCCCGGGCAAAGATGGTT

Mp313E CATGGCCATCATTCTGGTGACCACGCAAAAAGGAGCAGCCATTCCCGGGC-AAGATGGTT

Reference TCAGAAGCCAGCGAGCTTGGCATTGGCACACACTATACACACTGGTCGCTGATATTTCGA

Coding -------------------------------------------GGTCGCTGATATTTYGA

B73 TCAGAAGCCAGCGAGCTTGGCATTGGCACACACTATACACACTGGTCGCTGATATTTCGA

Va35 TCAGAAGCCAGCGAGCTTGGCATTGGCACACACTATACACACTGGTCGCTGATATTTCGA

T173 TCAGAAGCCAGCGAGCTTGGCATTGGCACACACTATACACACTGGTCGCTGATATTYTGA

Mp715 TCAGAAGCCAGCGAGCTTGGCATTGGCACACACTATACACACTGGTCGCTGATATTTTGA

Mp313E TCAGAAGCCAGCGAGCTTGGCATTGGCACACACTATACACACTGGTCGCTGATATTTTGA

Reference TTCCCCACGTGCGACACGGCATCTACCCGTCGAGGAACTCCAC----ATCTCTCCATGAA

Coding TTCCCCACGTGCGACACGGCATCTACCCGTCGAGGAACTCCAC----ATCTCTCCATGMA

B73 TTCCCCACGTGCGACACGGCATCTACCCGTCGAGGAACTCCAC----ATCTCTCCATGAA

Va35 TTCCCCACGTGCGACACGGCATCTACCCGTCGAGGAACTCCAC----ATCTCTCCATGAA

T173 TTCCCCACGTGCGACACGGCATCTACCCGTCGAGGAACTCCACRTCTATCTCTCCATGAA

Mp715 TTCCCCACGTGCGACACGGCATCTACCCGTCGAGGAACTCCACATCTATCTCTCCATGAA

Mp313E TTCCCCACGTGCGACACGGCATCTACCCGTCGAGGAACTCCACATCTATCTCTCCATGAA

Reference GCTCGCTATATATGCATGTGAGCTTGGCATTGGCACACCACAGAGCAGCCACTTGGATCC

Coding GCTCGCTAYATATGCATGTGAGCTTGGCATTGGCACACCACAGAGCAGCCACTTGGATYC

B73 GCTCGCTATATATGCATGTGAGCTTGGCATTGGCACACCACAGAGCAGCCACTTGGATCC

Va35 GCTCGCTATATATGCATGTGAGCTTGGCATTGGCACACCACAGAGCAGCCACTTGGATCC

T173 GCTCGCTATATATGCATGTGAGCTTGGCATTGGCACACCACAGAGCAGCCACTTGGATCC

Mp715 GCTCGCTATATATGCATGTGAGCTTGGCATTGGCACACCACAGAGCAGCCACTTGGATCC

Mp313E GCTCGCTATATATGCATGTGAGCTTGGCATTGGCACACCACAGAGCAGCCACTTGGATCC

Reference GGTGCTCAGCGGAACAGCTAGCTATAGCCTGTAGCTAGCAGCTAGCTTCAGTCACAGGCC

Coding GGTGCTCAGCGGAACAGCTAGCTATAGCCTGTAGCTAGCAGCTAGCTTCAGTCACAGGCC

B73 GGTGCTCAGCGGAACAGCTAGCTATAGCCTGTAGCTAGCAGCTAGCTTCAGTCACAGGCC

Va35 GGTGCTCAGCGGAACAGCTAGCTATAGCCTGTAGCTAGCAGCTAGCTTCAGTCACAGGCC

T173 GGTGCTCAGCGGAACAGCTAGCTATAGCCTGTAGCTAGCAGCTAGCTTCAGTCACAGGCC

Mp715 GGTGCTCAGCGGAACAGCTAGCTATAGCCTGTAGCTAGCAGCTAGCTTCAGTCACAGGCC

Mp313E GGTGCTCAGCGGAACAGCTAGCTATAGCCTGTAGCTAGCAGCTAGCTTCAGTCACAG-CA

Reference GGACGGTCGGCTCACACACAGCTAGCCGCCGCCGCCGCTAGCTTAGCCACCAGTAGTCCG

Coding GGACGGTCGGCTCACACACAGCTAGCCGCCGCCGCHGCTWGCTTAGCCACCAGTAGTCCG

B73 GGACGGTCGGCTCACACACAGCTAGCCGCCGCCGCCGCTAGCTTAGCCACCAGTAGTCCG

Va35 GGACGGTCGGCTCACACACAGCTAGCCGCCGCCGCTGCTAGCTTAGCCACCAGTAGTCCG

T173 GGACGGTCGGCTCACACACAGCTAGCCGCCGCCGCTGCTAGCTTAGCCACCAGTAGTCCG

Mp715 GGACGGTCGGCTCACACACAGCTAGCCGCCGCCGCTGCTAGCTTAGCCACCAGTAGTCCG

Mp313E GGACGGTCGGCT--CACACAGCTAGCCGCCGCCGCTGCTAGCTTAGCCACCAGTAGTCCG

Reference ATCTGATCTACAGGCA----------AGGGGCAACTAGCTAGCTAGCCGCGCGCCGGCGC

Coding ATCTRATCYWCAGGCA----------AGGGGCAACTAGCTAGCTAGCCGCGCGCCGGCGC

B73 ATCTGATCTACAGGCA----------AGGGGCAACTAGCTAGCTAGCCGCGCGCCGGCGC

Va35 ATCTGATCTACAGGCAGGGGGCCAGCAGGGGCAGCTAGCTAGCTAGCCGCGCGCCGGCGC

T173 ATCTGATCTACAGGCAGGGGGCCAGCAGGGGCAGCTAGCTAGCTAGCCGCGCGCCGGCGC

Mp715 ATCTGATCTACAGGCAGGGGGCCAGCAGGGGCAGCTAGCTAGCTAGCCGCGCGCCGGCGC

Mp313E ATCTGATCTACAGGCAGGGGGCCAGCAGGGGCAGCTAGCTAGCTAGCCGCGCGCCGGCGC

START CODON 1 2

Reference CATGATGAACCTGAACCTGAAGCAGCCTCTGGTGCTGCCCGCGCACCACAGCAATGTCGT

Coding CATGATGAACCTGAACCTGAAGCAGCCKCTSGTGCTGCCCGCGCACCACRGCAATGTCGT

B73 CATGATGAACCTGAACCTGAAGCAGCCTCTGGTGCTGCCCGCGCACCACAGCAATGTCGT

Va35 CATGATGAACCTGAACCTGAAGCAGCCTCTGGTGCTGCCCGCGCACCACAGCAATGTCGT

T173 CATGATGAACCTGAACCTGAAGCAGCCGCTCGTGCTGCCCGCGCACCACAGCAATGTCGT

Mp715 CATGATGAACCTGAACCTGAAGCAGCCGCTCGTGCTGCCCGCGCACCACAGCAATGTCGT

Mp313E CATGATGAACCTGAACCTGAAGCAGCCGCTCGTGCTGCCCGCGCACCACAGCAATGTCGT

3 4

Reference CGGCTCGCGCCTGTCGTCGTCGTCGCCCTCGGCAGCCGCCGCCAGCAGGAGGACCGGCGG

Coding CGGCTCGYGCCTRTCGTCGYYGTCGCCCTCGGCRGCCGCCGCCAGCAGGAGGACCGKCGG

B73 CGGCTCGCGCCTGTCGTCGTCGTCGCCCTCGGCAGCCGCCGCCAGCAGGAGGACCGGCGG

Va35 CGGCTCGCGCCTGTCGTCGCCGTCGCCCTCGGCGGCCGCCGCCAGCAGGAGGACCGGCGG

T173 CGGCTCGCGCCTGTCGTCGTCGTCGCCCTCGGCAGCCGCCGCCAGCAGGAGGACCGGCGG

Mp715 CGGCTCGCGCCTGTCGTCGTCGTCGCCCTCGGCAGCCGCCGCYAGCAGGAGGACCGGCGG

Mp313E CGGCTCGCGCCTGTCGTCGTCGTCGCCCTCGGCAGCCGCCGCCAGCAGGAGGACCGGCGG

5

Reference CGGCGTGTCCTCCCGGTCCGGCTCCCGGCGGCACGTGCGGCTGCCGAGGATCAGCTGCAG

Coding CGGCGTGTCCTCCCGGTCCGGCTCCCGGCGGCMCGTGCGGCTGCCGAGGATCAGCTGCAG

B73 CGGCGTGTCCTCCCGGTCCGGCTCCCGGCGGCACGTGCGGCTGCCGAGGATCAGCTGCAG

Va35 CGGCGTGTCCTCCCGGTCCGGCTCCCGGCGGCCCGTGCGGCTGCCGAGGATCAGCTGCAG

T173 CGGCGTGTCCTCCCGGTCCGGCTCCCGGCGGCACGTGCGGCTGCCGAGGATCAGCTGCAG

Mp715 CGGCGTGTCCTCCCGGTCCGGCTCCCGGCGGCACGTGCGGCTGCCGAGGATCAGCTGCAG

Mp313E CGGCGTGTCCTCCCGGTCCGGCTCCCGGCGGCACGTGCGGCTGCCGAGGATCAGCTGCAG

6

Reference CGCCACCGAGGAGGTCAGCGGCGCCGTGTCGTCCGTCACCGTGGAGAGGATGCTCACGGT

Coding CGCCACCGRGGAGGTCAGCGGCGCCGTGTCGTCCGTCMCMGTGGAGAGGATGCTSACGGT

B73 CGCCACCGAGGAGGTCAGCGGCGCCGTGTCGTCCGTCACCGTGGAGAGGATGCTCACGGT

Va35 CGCCACCGAGGAGGTCAGCGGCGCCGTGTCGTCCGTCACCGTGGAGAGGATGCTGACGGT

T173 CGCCACCGAGGAGGTCAGCGGCGCCGTGTCGTCCGTCACCGTGGAGAGGATGCTCACGGT

Mp715 CGCCACCGAGGAGGTCAGCGGCGCCGTGTCGTCCGTCACCGTGGAGAGGATGCTCACGGT

Mp313E CGCCACCGAGGAGGTCAGCGGCGCCGTGTCGTCCGTCACCGTGGAGAGGATGCTCACGGT

Reference GACGGCGTCGGTGGAGGCGTCGCCGGCCATCGGGCAGATGTACTTCCAGCGCGCCGTCGA

Coding GACGGCGTCGGTGGAGGCGTCGCCGGCCATCGGGCAGAYKTACTTCCAGCGCGCCGTCGA

B73 GACGGCGTCGGTGGAGGCGTCGCCGGCCATCGGGCAGATGTACTTCCAGCGCGCCGTCGA

Va35 GACGGCGTCGGTGGAGGCGTCGCCGGCCATCGGGCAGATGTACTTCCAGCGCGCCGTCGA

T173 GACGGCGTCGGTGGAGGCGTCGCCGGCCATCGGGCAGATGTACTTCCAGCGCGCCGTCGA

Mp715 GACGGCGTCGGTGGAGGCGTCGCCGGCCATCGGGCAGATGTACTTCCAGCGCGCCGTCGA

Mp313E GACGGCGTCGGTGGAGGCGTCGCCGGCCATCGGGCAGATGTACTTCCAGCGCGCCGTCGA

7 89 10

Reference CGACATCGGCGACCTCCTCGGCAAGACGCTGCTGCTCGAGCTCGTCAGCTCCGAGCTCGA

Coding CGACRTCGGCGACCTMYTCGGCAAGACGCTGCTGCTSGAGCTCGTCAGCTCCGAGCTCGA

B73 CGACATCGGCGACCTCCTCGGCAAGACGCTGCTGCTCGAGCTCGTCAGCTCCGAGCTCGA

Va35 CGACGTCGGCGACCTATTCGGCAAGACGCTGCTGCTGGAGCTCGTCAGCTCCGAGCTCGA

T173 CGACATCGGCGACCTCCTCGGCAAGACGCTGCTGCTCGAGCTCGTCAGCTCCGAGCTCGA

Mp715 CGACATCGGCGACCTCCTCGGCAAGACGCTGCTGCTCGAGCTCGTCAGCTCCGAGCTCGA

Mp313E CGACGTCGGCGACCTATTCGGCAAGACGCTGCTGCTGGAGCTCGTCAGCTCCGAGCTCGA

Reference CGCAAGTGAGTATAGTCACTCA----TACTGTCGGCCGTATGGATCGGATGGGCGGGCGC

Coding CGCAA-------------------------------------------------------

B73 CGCAA-------------------------------------------------------

Va35 CGCAA-------------------------------------------------------

T173 CGCAAGTGAGTATAGTCACTCA----TACTGTCGGCCGTATGGATGGGC--GCCATGCAT

Mp715 CGCAAGTGAGTATAGTCACTCA----TACTGTCGGCCGTATGGATGGGC--GCCATGCAT

Mp313E CGCAAGTGAGTATAGTCACTCACTCATACTGCTGTCCGTATGGATGGGC--GCCATGC--

Reference CATGCATGCATGCATCCTAGCTGTTAGTAGCTGAGCT--AGATCCAACAATAATGGTGGC

Coding ------------------------------------------------------------

B73 ------------------------------------------------------------

Va35 ------------------------------------------------------------

T173 GCATGATGCATGCATCCT-GCTAGTAGTAGCTGAGCT--AGATCCAACACTAATGGTGGC

Mp715 GCATGATGCATGCATCCT-GCTAGTAGTAGCTGAGCT--AGATCCAACACTAATGGTGGC

Mp313E -----ATGCATGCATCCTAACTGCTAGTAGCTGAGCTCTAGATCCAACAATAATGGTGGC

Reference TACTATAT--------------------ATCCATGGCCATTATATTGCTCA--CAGCGAC

Coding ------------------------------------------------------------

B73 ------------------------------------------------------------

Va35 ------------------------------------------------------------

T173 TACTATACTATATATCTATATATTAATGGGCCATGGCCATTATATTGCTCAGCCAGCAAC

Mp715 TACTATACTATATATCTATATATTAATGGGCCATGGCCATTATATTGCTCAGCCAGCAAC

Mp313E TACTATAT--------------------ATCCGTGGCCATTATATTGCTCA--CAGCGAC

Reference TAAACGAAAATCACTACTGAACCGTGGATTTATATATATATATATATATATATATATATA

Coding ------------------------------------------------------------

B73 ------------------------------------------------------------

Va35 ------------------------------------------------------------

T173 TAAACGAAAATCACTACTGAACCGTGG--------------------------------A

Mp715 TAAACGAAA-TCACTACTGAACCGTGG--------------------------------A

Mp313E TAAACGAAAATCACTACTGAACCGTGA--------------------------------A

Reference TATATATATAGATGTG-----TGGATGATGATTATCATAACAATAATGAGGGGTGAGAAT

Coding ------------------------------------------------------------

B73 ------------------------------------------------------------

Va35 ------------------------------------------------------------

T173 TATATCTATAGATGTG-----TGGATGATGGTTATCATAACAATAATG-------AGAAT

Mp715 TATATCTATAGATGTG-----TGGATGATGGTTATCATAACAATAATG-------AGAAT

Mp313E TTTATATATAGATGTGTGAAATGGATGATAGTTATCATAACAATAATG-------AGAAT

Reference ATAGTTCAAATATATGTGTGCAAGAAATATAGATGCTCTCCACAATAATGGACAAAAGGA

Coding ------------------------------------------------------------

B73 ------------------TGCAAGAAATATAGATGCTCTCCACAATAATGGACAAAAGGA

Va35 ------------------TGCAAGAAATATAGATGCTCTCCACAATAATGGACAAAAGGA

T173 ATAGTTCAAATATATGTGTGCAAGAAATATAGATGCTCTCCACAATAATGGACAAAAGGA

Mp715 ATAGTTCAAATATATGTGTGCAAGAAATATAGATGCTCTCCACAATAATGGACAAAAGGA

Mp313E ATAGTTCAAATATATGTGTGCAAGAAATATAGATGCTCTCCACAATAATGGACAAAAGGA

Reference CTTGAACTGCAAGACGCCATACCATACGCGTACGACTAGACGAGAGTTTGTCGTAGAAGA

Coding ------------------------------------------------------------

B73 CTTGAACTGCAAGACGCCATACCATACGCGTACGACTAGACGAGAGTTTGTCGTAGAAGA

Va35 CTTGAACTGCAAGACGCCATACCATACGCGTACGACTAGACGAGAGTTTGTCGTAGAAGA

T173 CTTGAACTGCAAGACGCCATACCATACGCGTACGACTAGACGAGAGTTTGTCGTAGAAGA

Mp715 CTTGAACTGCAAGACGCCATACCATACGCGTACGACTAGACGAGAGTTTGTCGTAGAAGA

Mp313E CTTGAACTGCAAGACGCCATACCATACGCGTACGACTAGACGAGAGTTTGTCGTAGAAGA

Reference CTACGCGCGTACGTACGCCGTAGAAAATAAAGGGAAAA-TCTGAGCTTGTGAGCATGCATG

Coding -------------------------------------------------------------

B73 CTACGCGCGTACGTACGCCGTAGAAAATAAAGGGAAAA-TCTGAGCTTGTGAGCATGCATG

Va35 CTACGCGCGTACGTA-------GGAAATAAAGGGAAAAATCTGAGCTAGTGAGCAT--ATR

T173 CTACGCGCGTAC----GCCGTAAGAAATAAAGGGAAAAATCTGAGCTAGTGAGCATGCATG

Mp715 CTACGCGCGTAC----GCCGTAAGAAATAAAGGGAAAAATCTGAGCTAGTGAGCATGCATG

Mp313E CTACGCGCGTACGTA-------GGAAATAAAGGGAAAAATCTGAGCTAGTGAGCATGCA--

11

Reference CATCATGCATGTGGTGTGGTGGTGCACTGCAGAGTCGGGCGTGGAGAAGACGCGGGTGAC

Coding --------------------------------ARTCGGGCGTGGAGAAGACGCGGGTGAC

B73 CATCATGCATGTGGTGTGGTGGTGCACTGCAGAGTCGGGCGTGGAGAAGACGCGGGTGAC

Va35 ---CATGCATGC-ATGTGGTGGTGCACTGCAGAGTCGGGCGTGGAGAAGACGCGGGTGAC

T173 ---CATGCAT---GTGTGGTGGTGCACTGCAGAGTCGGGCGTGGAGAAGACGCGGGTGAC

Mp715 C------------ATGTGGTGGTGCACTGCAGAGTCGGGCGTGGAGAAGACGCGGGTGAC

Mp313E ---TATGA-----ATGTGGCGGTGCACTGCAGAATCGGGCGTGGAGAAGACGCGGGTGAC

Reference GGCGTACGCGCACAAGACGCTGCGGGAGGGCCACTACGAGGCGGAGTTCAAGGTGCCGGC

Coding GGCGTACGCGCACAAGACGCTGCGGGAGGGCCACTACGAGGCGGAGTTCAWGGTGCCGGC

B73 GGCGTACGCGCACAAGACGCTGCGGGAGGGCCACTACGAGGCGGAGTTCAAGGTGCCGGC

Va35 GGCGTACGCGCACAAGACGCTGCGGGAGGGCCACTACGAGGCGGAGTTCAAGGTGCCGGC

T173 GGCGTACGCGCACAAGACGCTGCGGGAGGGCCACTACGAGGCGGAGTTCAAGGTGCCGGC

Mp715 GGCGTACGCGCACAAGACGCTGCGGGAGGGCCACTACGAGGCGGAGTTCAAGGTGCCGGC

Mp313E GGCGTACGCGCACAAGACGCTGCGGGAGGGCCACTACGAGGCGGAGTTCAAGGTGCCGGC

12 13

Reference GTCGTTCGGGCCGGTGGGCGCGGTGCTGGTGGAGAACGAGCACCACAAGGAGGTCTTCAT

Coding GTCGTTCGGKCYGGTGGGCGCGGTGCTGGTGGAGAAYGAGCACCACAAGGAGGTCTTCAT

B73 GTCGTTCGGGCCGGTGGGCGCGGTGCTGGTGGAGAACGAGCACCACAAGGAGGTCTTCAT

Va35 GTCGTTCGGGCTGGTGGGCGCGGTGCTGGTGGAGAACGAGCACCACAAGGAGGTCTTCAT

T173 GTCGTTCGGGCCGGTGGGCGCGGTGCTGGTGGAGAACGAGCACCACAAGGAGGTCTTCAT

Mp715 GTCGTTCGGGCCGGTGGGCGCGGTGCTGGTGGAGAACGAGCACCACAAGGAGGTCTTCAT

Mp313E GTCGTTCGGTCCGGTGGGCGCGGTGCTGGTGGAGAACGAGCACCACAAGGAGGTCTTCAT

Reference CAAGGAGATCAAGCTCGTCACCGGCGGCGACAGCAGCACCGCCGTCACCTTCGACTGCAA

Coding CAAGGAGATYAAGCTCGTCACCGGCGGCGACAGCAGCACCGCCGTCACCTTCGACTGCAA

B73 CAAGGAGATCAAGCTCGTCACCGGCGGCGACAGCAGCACCGCCGTCACCTTCGACTGCAA

Va35 CAAGGAGATCAAGCTCGTCACCGGCGGCGACAGCAGCACCGCCGTCACCTTCGACTGCAA

T173 CAAGGAGATCAAGCTCGTCACCGGCGGCGACAGCAGCACCGCCGTCACCTTCGACTGCAA

Mp715 CAAGGAGATCAAGCTCGTCACCGGCGGCGACAGCAGCACCGCCGTCACCTTCGACTGCAA

Mp313E CAAGGAGATCAAGCTCGTCACCGGCGGCGACAGCAGCACCGCCGTCACCTTCGACTGCAA

Reference CTCCTGGGTGCACTCCAAGTTCGACAACCCGGAGAAGCGCATCTTCTTCACCCTCAAGGT

Coding CTCCTGGGTGCACTCCAAGTTCGACAACCCGGAGAAGCGCATCTTCTTCACCCTCAAG--

B73 CTCCTGGGTGCACTCCAAGTTCGACAACCCGGAGAAGCGCATCTTCTTCACCCTCAAGGT

Va35 CTCCTGGGTGCACTCCAAGTTCGACAACCCGGAGAAGCGCATCTTCTTCACCCTCAAGGT

T173 CTCCTGGGTGCACTCCAAGTTCGACAACCCGGAGAAGCGCATCTTCTTCACCCTCAAGGT

Mp715 CTCCTGGGTGCACTCCAAGTTCGACAACCCGGAGAAGCGCATCTTCTTCACCCTCAAGGT

Mp313E CTCCTGGGTGCACTCCAAGTTCGACAACCCGGAGAAGCGCATCTTCTTCACCCTCAAGGT

Reference ACGTACGTCGATCAGCATG----GTGACCGCGTGCATGCAT--GTATGCTG-----ACTG

Coding ------------------------------------------------------------

B73 ACGTACGTCGATCAGCATG----GTGACCGCGTGCATGCAT--GTATGCTG-----ACTG

Va35 ACGTCGGTTAGCGTGCATG----GTGACTGCGTGCGTGCATATGTATGCTG-----ACTG

T173 ACGTACGTCGATCAGCATGCATGGTGACTGCGTGCGTGCATATGTATGCTGTGCTGACTG

Mp715 ACGTCGGTTAGCGTGCATG----GTGACTGCGTGCGTGCATATGTATGCTG-----ACTG

Mp313E ACGTCGGTTAGCGTGCATG----GTGACTGCGTGCGTGCATATGTATGCTG-----ACTG

Reference ACACTCACGGCTGCT---------TACTTTTCTGTGCAGTCATACCTGCCGTCCGACACG

Coding ---------------------------------------TCATACCTGCCGTCCGACACG

B73 ACACTCACGGCTGCT---------TACTTTTCTGTGCAGTCATACCTGCCGTCCGACACG

Va35 ACACTCACGGCTGCT---------TACTTTTCTGTGCAGTCATACCTGCCGTCCGACACG

T173 ACACTCACGGCTGCTGCTGCTGCTTACTTTTCTGTGCAGTCATACCTGCCGTCCGACACG

Mp715 ACACTCACGGCTGCT---------TACTTTTCTGTGCAGTCATACCTGCCGTCCGACACG

Mp313E ACACTCACGGCTGCT---------TACTTTTCTGTGCAGTCATACCTGCCGTCCGACACG

Reference CCCAAGGGGCTGGAGGACCTGAGGAAGAAGGACCTGCAGGCGCTGCGCGGCGACGGGCAC

Coding CCCAAGGGGCTGGARGACCTGASGAAGAAGGACCTGCAGGCGCTGCGCGGCGACGGGCAC

B73 CCCAAGGGGCTGGAGGACCTGAGGAAGAAGGACCTGCAGGCGCTGCGCGGCGACGGGCAC

Va35 CCCAAGGGGCTGGAGGACCTGAGGAAGAAGGACCTGCAGGCGCTGCGCGGCGACGGGCAC

T173 CCCAAGGGGCTGGAGGACCTGAGGAAGAAGGACCTGCAGGCGCTGCGCGGCGACGGGCAC

Mp715 CCCAAGGGGCTGGAGGACCTGAGGAAGAAGGACCTGCAGGCGCTGCGCGGCGACGGGCAC

Mp313E CCCAAGGGGCTGGAGGACCTGAGGAAGAAGGACCTGCAGGCGCTGCGCGGCGACGGGCAC

Reference GGCGAGCGCAAGGTGTTCGAGCGCGTCTACGACTACGACGTGTACAACGACCTGGGCGAC

Coding GGCGAGCGCAAGGTGTTCGARCGCGTCTACGACTACGACGTGTACAACGACCTGGGCGAC

B73 GGCGAGCGCAAGGTGTTCGAGCGCGTCTACGACTACGACGTGTACAACGACCTGGGCGAC

Va35 GGCGAGCGCAAGGTGTTCGAGCGCGTCTACGACTACGACGTGTACAACGACCTGGGCGAC

T173 GGCGAGCGCAAGGTGTTCGAGCGCGTCTACGACTACGACGTGTACAACGACCTGGGCGAC

Mp715 GGCGAGCGCAAGGTGTTCGAGCGCGTCTACGACTACGACGTGTACAACGACCTGGGCGAC

Mp313E GGCGAGCGCAAGGTGTTCGAGCGCGTCTACGACTACGACGTGTACAACGACCTGGGCGAC

Reference CCGGACAAGAACCCGGCCCACCAGCGGCCCGTGCTGGGCGGCAACAAGCAGTACCCATAC

Coding CCGGACAAGAACCCGGCCCACCAGCGGCCCGTGCTGGGCGGCAACAAGCAGTACYYATAC

B73 CCGGACAAGAACCCGGCCCACCAGCGGCCCGTGCTGGGCGGCAACAAGCAGTACCCATAC

Va35 CCGGACAAGAACCCGGCCCACCAGCGGCCCGTGCTGGGCGGCAACAAGCAGTACCCATAC

T173 CCGGACAAGAACCCGGCCCACCAGCGGCCCGTGCTGGGCGGCAACAAGCAGTACCCATAC

Mp715 CCGGACAAGAACCCGGCCCACCAGCGGCCCGTGCTGGGCGGCAACAAGCAGTACCCATAC

Mp313E CCGGACAAGAACCCGGCCCACCAGCGGCCCGTGCTGGGCGGCAACAAGCAGTACCCATAC

Reference CCGCGCCGCTGCCGCACCGGCCGCCCCAGGACCAAGAAGGACCCCGAGACGGAGATGCGC

Coding CCGCGCCGCTGCCGCACCGGCCGCCCCAGGACCAAGAAGGACCCCGAGACGGAGATGCGC

B73 CCGCGCCGCTGCCGCACCGGCCGCCCCAGGACCAAGAAGGACCCCGAGACGGAGATGCGC

Va35 CCGCGCCGCTGCCGCACCGGCCGCCCCAGGACCAAGAAGGACCCCGAGACGGAGATGCGC

T173 CCGCGCCGCTGCCGCACCGGCCGCCCCAGGACCAAGAAGGACCCCGAGACGGAGATGCGC

Mp715 CCGCGCCGCTGCCGCACCGGCCGCCCCAGGACCAAGAAGGACCCCGAGACGGAGATGCGC

Mp313E CCGCGCCGCTGCCGCACCGGCCGCCCCAGGACCAAGAAGGACCCCGAGACGGAGATGCGC

14

Reference GAGGGCCACAACTACGTGCCCCGCGACGAGCAGTTCTCGGAGGTGAAGCAGCTCACGTTC

Coding RAGGGCCACRRCTACGTGCCCCGCRACGAGCAGTTCTCGGAGGTGAAGCAGCTCACGTTC

B73 GAGGGCCACAACTACGTGCCCCGCGACGAGCAGTTCTCGGAGGTGAAGCAGCTCACGTTC

Va35 AAGGGCCACAACTACGTGCCCCGCGACGAGCAGTTCTCGGAGGTGAAGCAGCTCACGTTC

T173 GAGGGCCACAACTACGTGCCCCGCGACGAGCAGTTCTCGGAGGTGAAGCAGCTCACGTTC

Mp715 GAGGGCCACAACTACGTGCCCCGCGACGAGCAGTTCTCGGAGGTGAAGCAGCTCACGTTC

Mp313E GAGGGCCACAACTACGTGCCCCGCGACGAGCAGTTCTCGGAGGTGAAGCAGCTCACGTTC

Reference GGGGCCACCACGCTGCGCTCCGGCCTGCACGCGCTGCTGCCGGCGCTCCGCCCGCTGCTC

Coding GGGGCYACCACGCTGCGCTCCGGCCTGCACGCGCTGCTGCCGGCGCTCCGCCCGCTGCTC

B73 GGGGCCACCACGCTGCGCTCCGGCCTGCACGCGCTGCTGCCGGCGCTCCGCCCGCTGCTC

Va35 GGGGCCACCACGCTGCGCTCCGGCCTGCACGCGCTGCTGCCGGCGCTCCGCCCGCTGCTC

T173 GGGGCCACCACGCTGCGCTCCGGCCTGCACGCGCTGCTGCCGGCGCTCCGCCCGCTGCTC

Mp715 GGGGCCACCACGCTGCGCTCCGGCCTGCACGCGCTGCTGCCGGCGCTCCGCCCGCTGCTC

Mp313E GGGGCCACCACGCTGCGCTCCGGCCTGCACGCGCTGCTGCCGGCGCTCCGCCCGCTGCTC

15 16

Reference ATCAACAAGAAGGATCTGCGCTTCCCGCACTTCCCCGCCATCGACGACCTCTTCAGCGAC

Coding ATCAACAAGAAGGATCTGCGCTTCCCGCACTTYCCCGCCATCGACGAMCTCTWCAGCGAC

B73 ATCAACAAGAAGGATCTGCGCTTCCCGCACTTCCCCGCCATCGACGACCTCTTCAGCGAC

Va35 ATCAACAAGAAGGATCTGCGCTTCCCGCACTTCCCCGCCATCGACGAACTCTACAGCGAC

T173 ATCAACAAGAAGGATCTGCGCTTCCCGCACTTCCCCGCCATCGACGAACTCTACAGCGAC

Mp715 ATCAACAAGAAGGATCTGCGCTTCCCGCACTTCCCCGCCATCGACGACCTCTTCAGCGAC

Mp313E ATCAACAAGAAGGATCTGCGCTTCCCGCACTTCCCCGCCATCGACGAACTCTACAGCGAC

17

Reference GGCATCCCGCTGCCGGCGCAGACCGGGTTCGACGCCATCCGCACCGTCGTCCCGCGCATG

Coding GGCATCCCGCTGCCGSCGCAGACCGGGTTCGACGCCATCCGCACCGTCGTCCCGCGCATG

B73 GGCATCCCGCTGCCGGCGCAGACCGGGTTCGACGCCATCCGCACCGTCGTCCCGCGCATG

Va35 GGCATCCCGCTGCCGGCGCAGACCGGGTTCGACGCCATCCGCACCGTCGTCCCGCGCATG

T173 GGCATCCCGCTGCCGCCGCAGACCGGGTTCGACGCCATCCGCACCGTCGTCCCGCGCATG

Mp715 GGCATCCCGCTGCCGGCGCAGACCGGGTTCGACGCCATCCGCACCGTCGTCCCGCGCATG

Mp313E GGCATCCCGCTGCCGGCGCAGACCGGGTTCGACGCCATCCGCACCGTCGTCCCGCGCATG

Reference GTCAAGCTGGTGGAGGACACCACCGACCACGTCCTCCGCTTCGAGGTGCCGGAGATGATA

Coding GTCAAGCTGGTGGAGGACACCACCGACCACGTCCTCCGCTTCGAGGTGCCGGAGATGATA

B73 GTCAAGCTGGTGGAGGACACCACCGACCACGTCCTCCGCTTCGAGGTGCCGGAGATGATA

Va35 GTCAAGCTGGTGGAGGACACCACCGACCACGTCCTCCGCTTCGAGGTGCCGGAGATGATA

T173 GTCAAGCTGGTGGAGGACACCACCGACCACGTCCTCCGCTTCGAGGTGCCGGAGATGATA

Mp715 GTCAAGCTGGTGGAGGACACCACCGACCACGTCCTCCGCTTCGAGGTGCCGGAGATGATA

Mp313E GTCAAGCTGGTGGAGGACACCACCGACCACGTCCTCCGCTTCGAGGTGCCGGAGATGATA

Reference GAGAGTAAGCTAGCTAGCAGACGCAC--TCATCTCAGGACTGCTTGTTATCTTAATCTTA

Coding GAGA--------------------------------------------------------

B73 GAGAGTAAGCTAGCTAGCAGACGCAC--TCATCTCAGGACTGCTTGTTATCTTAATCTTA

Va35 GAGAGTAAGCTAGCTAGAAGACACACACTCATCTCAGGACTGCTTGTTATCTTAATCTTA

T173 GAGAGTAAGCTAGCTAGAAGACACACACTCATCTCAGGACTGCTTGTTATCTTAATCTTA

Mp715 GAGAGTAAGCTAGCTAGAAGACACACACTCATCTCAGGACTGCTTGTTATCTTAATCTTA

Mp313E GAGAGTAAGCTAGCTAGAAGACACACACTCATCTCAGGACTGCTTGTTATCTTAATCTTA

Reference AAAAAAATAAATGATTTGCTTTGCTAGGGGACCGGTTCTCGTGGTTCAAGGACGAGGAGT

Coding ---------------------------GGGACCGGTTCTCGTGGTTCAAGGACGARGAGT

B73 AAAAAAATAAATGATTTGCTTTGCTAGGGGACCGGTTCTCGTGGTTCAAGGACGAGGAGT

Va35 AAAAAATWAAATCATTTGCTTTGCCAGGGGACCGGTTCTCGTGGTTCAAGGACGAGGAGT

T173 AAAAA---AAATCATTTGCTTTGCCAGGGGACCGGTTCTCGTGGTTCAAGGACGAGGAGT

Mp715 AAAAAATWAAATCATTTGCTTTGCCAGGGGACCGGTTCTCGTGGTTCAAGGACGAGGAGT

Mp313E AAAAAATWAAATCATTTGCTTTGCCAGGGGACCGGTTCTCGTGGTTCAAGGACGAGGAGT

18

Reference TCGCGAGGCAGACGATCGCGGGGCTCAACCCGCTGTGCATCCAGCTGCTGACTGAGTTCC

Coding TCGCGAGGCAGACGATCGCGGGGCTCAACCCGCTGTGCATCCAGCTGCTGACYGAGTTCC

B73 TCGCGAGGCAGACGATCGCGGGGCTCAACCCGCTGTGCATCCAGCTGCTGACTGAGTTCC

Va35 TCGCGAGGCAGACGATCGCGGGGCTCAACCCGCTGTGCATCCAGCTGCTGACCGAGTTCC

T173 TCGCGAGGCAGACGATCGCGGGGCTCAACCCGCTGTGCATCCAGCTGCTGACTGAGTTCC

Mp715 TCGCGAGGCAGACGATCGCGGGGCTCAACCCGCTGTGCATCCAGCTGCTGACCGAGTTCC

Mp313E TCGCGAGGCAGACGATCGCGGGGCTCAACCCGCTGTGCATCCAGCTGCTGACCGAGTTCC

Reference CCATCAAGAGCAAGCTGGACCCGGAGGTGTACGGGCCAGCGGAGTCCGCCATCACCAAGG

Coding CCRTCAAGAGCAAGCTGGACCCGGAGGTRTACRGGCCAGCRGAGTCCGCCATCACCAAGG

B73 CCATCAAGAGCAAGCTGGACCCGGAGGTGTACGGGCCAGCGGAGTCCGCCATCACCAAGG

Va35 CCATCAAGAGCAAGCTGGACCCGGAGGTGTACGGGCCAGCGGAGTCCGCCATCACCAAGG

T173 CCATCAAGAGCAAGCTGGACCCGGAGGTGTACGGGCCAGCGGAGTCCGCCATCACCAAGG

Mp715 CCATCAAGAGCAAGCTGGACCCGGAGGTGTACGGGCCAGCGGAGTCCGCCATCACCAAGG

Mp313E CCATCAAGAGCAAGCTGGACCCGGAGGTGTACGGGCCAGCGGAGTCCGCCATCACCAAGG

Reference AGATCCTGGAGAAGCAGATGAACGGCGCGCTGACCGTGGAGCAGGCGCTGGCGGCGAAGC

Coding AGRTCCTGGAGAAGCAGATGAACKGCGCGCTGACCGTGGAGCAGGCGCTGGCGGCGAAGC

B73 AGATCCTGGAGAAGCAGATGAACGGCGCGCTGACCGTGGAGCAGGCGCTGGCGGCGAAGC

Va35 AGATCCTGGAGAAGCAGATGAACGGCGCGCTGACCGTGGAGCAGGCGCTGGCGGCGAAGC

T173 AGATCCTGGAGAAGCAGATGAACGGCGCGCTGACCGTGGAGCAGGCGCTGGCGGCGAAGC

Mp715 AGATCCTGGAGAAGCAGATGAACGGCGCGCTGACCGTGGAGCAGGCGCTGGCGGCGAAGC

Mp313E AGATCCTGGAGAAGCAGATGAACGGCGCGCTGACCGTGGAGCAGGCGCTGGCGGCGAAGC

Reference GGCTGTTCATCCTGGACTACCACGACGTGTTCCTGCCCTACGTGCACAAGGTGCGGGAGC

Coding GGCTGTTCATCCTGGACTACCACGACGTRTTCCTGCCCTACGTGCACAAGGTGCGGGAGC

B73 GGCTGTTCATCCTGGACTACCACGACGTGTTCCTGCCCTACGTGCACAAGGTGCGGGAGC

Va35 GGCTGTTCATCCTGGACTACCACGACGTGTTCCTGCCCTACGTGCACAAGGTGCGGGAGC

T173 GGCTGTTCATCCTGGACTACCACGACGTGTTCCTGCCCTACGTGCACAAGGTGCGGGAGC

Mp715 GGCTGTTCATCCTGGACTACCACGACGTGTTCCTGCCCTACGTGCACAAGGTGCGGGAGC

Mp313E GGCTGTTCATCCTGGACTACCACGACGTGTTCCTGCCCTACGTGCACAAGGTGCGGGAGC

Reference TGCAGGACGCGACGCTCTACGCCTCGCGCACCATCTTCTTCCTGACGGACCTGGGCACGC

Coding TGCRGGACKCGACGCTCTACGCCTCGCGCACCRTCTTCTTCCTGACGGACCTGGGCACGC

B73 TGCAGGACGCGACGCTCTACGCCTCGCGCACCATCTTCTTCCTGACGGACCTGGGCACGC

Va35 TGCAGGACGCGACGCTCTACGCCTCGCGCACCATCTTCTTCCTGACGGACCTGGGCACGC

T173 TGCAGGACGCGACGCTCTACGCCTCGCGCACCATCTTCTTCCTGACGGACCTGGGCACGC

Mp715 TGCAGGACGCGACGCTCTACGCCTCGCGCACCATCTTCTTCCTGACGGACCTGGGCACGC

Mp313E TGCAGGACGCGACGCTCTACGCCTCGCGCACCATCTTCTTCCTGACGGACCTGGGCACGC

Reference TGATGCCGCTGGCCATCGAGCTGACGCGGCCCAAGTCGCCGACGCGGCCGCAGTGGAAGC

Coding TGATGCCGCTGGCCATCGAGCTGACGCGGCCCAAGTCGCCGACRCGGCCGCAGTGGAAGC

B73 TGATGCCGCTGGCCATCGAGCTGACGCGGCCCAAGTCGCCGACGCGGCCGCAGTGGAAGC

Va35 TGATGCCGCTGGCCATCGAGCTGACGCGGCCCAAGTCGCCGACGCGGCCGCAGTGGAAGC

T173 TGATGCCGCTGGCCATCGAGCTGACGCGGCCCAAGTCGCCGACGCGGCCGCAGTGGAAGC

Mp715 TGATGCCGCTGGCCATCGAGCTGACGCGGCCCAAGTCGCCGACGCGGCCGCAGTGGAAGC

Mp313E TGATGCCGCTGGCCATCGAGCTGACGCGGCCCAAGTCGCCGACGCGGCCGCAGTGGAAGC

19 20

Reference GGGCGTTCACGCACGGGCCCGACGCCACCGACGCCTGGCTGTGGAAGCTGGCCAAGGCGC

Coding GRGCGTTCACGCACGGGCCCGACGCCACCGACGCMTGGCTGTGGAAGCTGGCCAAGGCGC

B73 GGGCGTTCACGCACGGGCCCGACGCCACCGACGCCTGGCTGTGGAAGCTGGCCAAGGCGC

Va35 GGGCGTTCACGCACGGGCCCGACGCCACCGACGCATGGCTGTGGAAGCTGGCCAAGGCGC

T173 GGGCGTTCACGCACGGGCCCGACGCCACCGACGCCTGGCTGTGGAAGCTGGCCAAGGCGC

Mp715 GGGCGTTCACGCACGGGCCCGACGCCACCGACGCCTGGCTGTGGAAGCTGGCCAAGGCGC

Mp313E GAGCGTTCACGCACGGGCCCGACGCCACCGACGCCTGGCTGTGGAAGCTGGCCAAGGCGC

Reference ACGTGCTGACCCACGACACGGGGTACCACCAGCTGGTGAGCCACTGGCTGCGCACGCACT

Coding ACGTGCTGACCCACGACACGGGGTACCACCAGCTGGTGAGCCACTGGCTGCGCACGCACT

B73 ACGTGCTGACCCACGACACGGGGTACCACCAGCTGGTGAGCCACTGGCTGCGCACGCACT

Va35 ACGTGCTGACCCACGACACGGGGTACCACCAGCTGGTGAGCCACTGGCTGCGCACGCACT

T173 ACGTGCTGACCCACGACACGGGGTACCACCAGCTGGTGAGCCACTGGCTGCGCACGCACT

Mp715 ACGTGCTGACCCACGACACGGGGTACCACCAGCTGGTGAGCCACTGGCTGCGCACGCACT

Mp313E ACGTGCTGACCCACGACACGGGGTACCACCAGCTGGTGAGCCACTGGCTGCGCACGCACT

Reference GCTGCGTGGAGCCCTACATCATCGCCGCCAACCGGCAGCTGAGCCGGCTGCACCCGGTGT

Coding GCTGCGTGGAGCCCTACATCATCGCCGCCAACCGGCAGCTGAGCCGGCTGCACCCGGTGT

B73 GCTGCGTGGAGCCCTACATCATCGCCGCCAACCGGCAGCTGAGCCGGCTGCACCCGGTGT

Va35 GCTGCGTGGAGCCCTACATCATCGCCGCCAACCGGCAGCTGAGCCGGCTGCACCCGGTGT

T173 GCTGCGTGGAGCCCTACATCATCGCCGCCAACCGGCAGCTGAGCCGGCTGCACCCGGTGT

Mp715 GCTGCGTGGAGCCCTACATCATCGCCGCCAACCGGCAGCTGAGCCGGCTGCACCCGGTGT

Mp313E GCTGCGTGGAGCCCTACATCATCGCCGCCAACCGGCAGCTGAGCCGGCTGCACCCGGTGT

Reference ACCGCCTGCTGCACCCGCACTTCCGCTACACCATGGAGATCAACGCGCTGGCCAGGGAGG

Coding ACCGCCTGCTGCACCCGCACTTCCGCTACACCATGGAGATCAACGCGCTGGCCAGGGAGG

B73 ACCGCCTGCTGCACCCGCACTTCCGCTACACCATGGAGATCAACGCGCTGGCCAGGGAGG

Va35 ACCGCCTGCTGCACCCGCACTTCCGCTACACCATGGAGATCAACGCGCTGGCCAGGGAGG

T173 ACCGCCTGCTGCACCCGCACTTCCGCTACACCATGGAGATCAACGCGCTGGCCAGGGAGG

Mp715 ACCGCCTGCTGCACCCGCACTTCCGCTACACCATGGAGATCAACGCGCTGGCCAGGGAGG

Mp313E ACCGCCTGCTGCACCCGCACTTCCGCTACACCATGGAGATCAACGCGCTGGCCAGGGAGG

21

Reference CGCTCATCAACGCCGACGGCATCATCGAGGAGTCCTTCTGGCCGGGCAAGTACGCCGTCG

Coding CGCTCATCAACGCCGACGGCATCATCGAGGAGTCCTTCTGGCCGGGMAAGTACGCCGTCG

B73 CGCTCATCAACGCCGACGGCATCATCGAGGAGTCCTTCTGGCCGGGCAAGTACGCCGTCG

Va35 CGCTCATCAACGCCGACGGCATCATCGAGGAGTCCTTCTGGCCGGGAAAGTACGCCGTCG

T173 CGCTCATCAACGCCGACGGCATCATCGAGGAGTCCTTCTGGCCGGGCAAGTACGCCGTCG

Mp715 CGCTCATCAACGCCGACGGCATCATCGAGGAGTCCTTCTGGCCGGGCAAGTACGCCGTCG

Mp313E CGCTCATCAACGCCGACGGCATCATCGAGGAGTCCTTCTGGCCGGGCAAGTACGCCGTCG

22

Reference AGCTCAGCTCCGTGGCGTACGGCGCGACGTGGCAGTTCGACACGGAGGCGCTGCCCAACG

Coding AGCTYAGCTCCGTGGCGTACGGCGCGACGTGGCAGTTCGACACGGAGGCGCTGCCCAACG

B73 AGCTCAGCTCCGTGGCGTACGGCGCGACGTGGCAGTTCGACACGGAGGCGCTGCCCAACG

Va35 AGCTCAGCTCCGTGGCGTACGGCGCGACGTGGCAGTTCGACACGGAGGCGCTGCCCAACG

T173 AGCTTAGCTCCGTGGCGTACGGCGCGACGTGGCAGTTCGACACGGAGGCGCTGCCCAACG

Mp715 AGCTTAGCTCCGTGGCGTACGGCGCGACGTGGCAGTTCGACACGGAGGCGCTGCCCAACG

Mp313E AGCTCAGCTCCGTGGCGTACGGCGCGACGTGGCAGTTCGACACGGAGGCGCTGCCCAACG

23

Reference ACCTCATCAAGCGCGGGCTGGCCGTGCGCGGGGAGGACGGGGAGCTGGAGCTCACCATCA

Coding ACCTCRTCAAGCGCGGGCTGGCCGTGCGCGGGGAGGACGGGGAGCTGGAGCTCACCATCA

B73 ACCTCATCAAGCGCGGGCTGGCCGTGCGCGGGGAGGACGGGGAGCTGGAGCTCACCATCA

Va35 ACCTCGTCAAGCGCGGGCTGGCCGTGCGCGGGGAGGACGGGGAGCTGGAGCTCACCATCA

T173 ACCTCGTCAAGCGCGGGCTGGCCGTGCGCGGGGAGGACGGGGAGCTGGAGCTCACCATCA

Mp715 ACCTCGTCAAGCGCGGGCTGGCCGTGCGCGGGGAGGACGGGGAGCTGGAGCTCACCATCA

Mp313E ACCTCATCAAGCGCGGGCTGGCCGTGCGCGGGGAGGACGGGGAGCTGGAGCTCACCATCA

24

Reference AGGACTACCCCTACGCCCACGACGGGCTCCTGGTCTGGGACTCCATCAGGCAGTGGGCGT

Coding AGGACTACCCCTACGCCCACGACGGGCTCCTGGTCTGGGACTCCATCARGCAGTGGGCGT

B73 AGGACTACCCCTACGCSCACGACGGGCTCMTGGTCTGGRACWCCATMAGGCAGTGGGCGK

Va35 AGGACTACCCCTACGCCCACGACGGGCTCCTGGTCTGGGACTCCATCAAGCAGTGGGCGT

T173 AGGACTACCCCTACGCCCACGACGGGCTCCTGGTCTGGGACTCCATCAAGCAGTGGGCGT

Mp715 AGGACTACCCCTACGCCCACGACGGGCTCCTGGTCTGGGACTCCATCAAGCAGTGGGCGT

Mp313E AGGACTACCCCTACGCCCACGACGGGCTCCTGGTCTGGGACTCCATCAAGCAGTGGGCGT

Reference CCGAGTACGTCAACGTCTACTACAAGTCCGACGAGGCCGTGGCCGCCGACCCCGAGCTGA

Coding CCGAGTACGTCAACGTCTACTACAAGTCCGACGAGGCCGTGGCMGCCGACCCCGAGCTGA

B73 CCGASTACGTCAACGTCTACTACAAGTCCGACGAGGCCGTSGCCGCCGACCCCGAGCTGA

Va35 CCGAGTACGTCAACGTCTACTACAAGTCCGACGAGGCCGTGGCCGCCGACCCCGAGCTGA

T173 CCGAGTACGTCAACGTCTACTACAAGTCCGACGAGGCCGTGGCCGCCGACCCCGAGCTGA

Mp715 CCGAGTACGTCAACGTCTACTACAAGTCCGACGAGGCCGTGGCCGCCGACCCCGAGCTGA

Mp313E CCGAGTACGTCAACGTCTACTACAAGTCCGACGAGGCCGTGGCCGCCGACCCCGAGCTGA

25

Reference GGGCGTTCTGGGACGAGGTGCGCAACGTGGGGCACGGCGACAAGAAGGACGAGCCGTGGT

Coding GRGCGTTCTGGGACGAGGTGCGCAACGTGGGGCACGGCGACAAGAAGGACGAGCCGTGGT

B73 RGGCGTTCTGGGACGAGGTGCGCAACGTGGGSCACGGCGACAAGAAGGACGAGCCGTGGT

Va35 GGGCGTTCTGGGACGAGGTGCGCAACGTGGGGCACGGCGACAAGAAGGACGAGCCGTGGT

T173 GGGCGTTCTGGGACGAGGTGCGCAACGTGGGGCACGGCGACAAGAAGGACGAGCCGTGGT

Mp715 GGGCGTTCTGGGACGAGGTGCGCAACGTGGGGCACGGCGACAAGAAGGACGAGCCGTGGT

Mp313E GAGCGTTCTGGGACGAGGTGCGCAACGTGGGGCACGGCGACAAGAAGGACGAGCCGTGGT

Reference GGCCCGTGCTGGACACCCGCGACAGCCTGGTGGAGACGCTGACCACCATCATGTGGGTCA

Coding GGCCCGTGCTGGACACCCGCGACAGCCTGGTGGAGACGCTGACCACCATCATGTGGGTCA

B73 GGCCCGTGCTSGACACCCGCGACAGCCTSGYGGAGACGCTSACCACCATCATGTGGGTCA

Va35 GGCCCGTGCTGGACACCCGCGACAGCCTGGTGGAGACGCTGACCACCATCATGTGGGTCA

T173 GGCCCGTGCTGGACACCCGCGACAGCCTGGTGGAGACGCTGACCACCATCATGTGGGTCA

Mp715 GGCCCGTGCTGGACACCCGCGACAGCCTGGTGGAGACGCTGACCACCATCATGTGGGTCA

Mp313E GGCCCGTGCTGGACACCCGCGACAGCCTGGTGGAGACGCTGACCACCATCATGTGGGTCA

Reference CCTCCGGCCACCACTCGGCCGTCAACTTCGGCCAGTACCACTTCGCCGGCTACTTCCCCA

Coding CCTCCGGCCACCACTCGGCCGTCAACTTCGGSCAGTACCACTTCGCCGGCTACTTCCCCA

B73 CCTCCGGCCACCACTCGGCSGTCAACTTCGGCCAGTACCACTTCGCCGGCTACTTCCCCA

Va35 CCTCCGGCCACCACTCGGCCGTCAACTTCGGCCAGTACCACTTCGCCGGCTACTTCCCCA

T173 CCTCCGGCCACCACTCGGCCGTCAACTTCGGSCAGTACCACTTCGCCGGCTACTTCCCCA

Mp715 CCTCCGGCCACCACTCGGCCGTCAACTTCGGSCAGTACCACTTCGCCGGCTACTTCCCCA

Mp313E CCTCCGGCCACCACTCGGCYGTCAACTTCGGCCAGTACCACTTCGCCGGCTACTTCCCCA

26

Reference ACCGGCCGACCACCATCCGGAAGAACATGCCGGTGGAGGAGGGCGGGCCGGGCGAGGAGA

Coding ACCGGCCGACCACCATCCGGAAGAACATGCCGGTGGAGGAGGGCGGGSCGGGCGRGGAGA

B73 ACCGGCCGACCACCATCCGGAAGAACATGCCGGTGGAGGAGGGCGGGCCGGGCGAGGAGA

Va35 ACCGGCCGACCACCATCCGGAAGAACATGCCGGTGGAGGAGGGCGGGCCGGGCGGGGAGA

T173 ACCGGCCGACCACCATCCGGAAGAACATGCCGGTGGAGGAGGGCGGGCCGGGCGAGGAGA

Mp715 ACCGGCCGACCACCATCCGGAAGAACATGCCGGTGGAGGAGGGCGGGCCGGGCGAGGAGA

Mp313E ACCGGCCGACCACCATCCGGAAGAACATGCCGGTGGAGGAGGGCGGGCCGGGCGGGGAGA

Reference TGGAGAAGTTCCTCAAGCAGCCGGAGACGACGCTGCTGGACATGCTGCCCACGCAGATGC

Coding TGGAGAAGTTCCTCAAGCAGCCGGAGACGACGCTGCTGGACATGCTGCCCACGCAGATGC

B73 TGGAGAAGTTCCTCAAGCAGCCGGAGACGACGCTGCTGGACATGCTGCCCACGCAGATGC

Va35 TGGAGAAGTTCCTCAAGCAGCCGGAGACGACGCTGCTGGACATGCTGCCCACGCAGATGC

T173 TGGAGAAGTTCCTCAAGCAGCCGGAGACGACGCTGCTGGACATGCTGCCCACGCAGATGC

Mp715 TGGAGAAGTTCCTCAAGCAGCCGGAGACGACGCTGCTGGACATGCTGCCCACGCAGATGC

Mp313E TGGAGAAGTTCCTCAAGCAGCCGGAGACGACGCTGCTGGACATGCTGCCCACGCAGATGC

Reference AGGCCATCAAGGTCATGACGACGCTGGACATCCTCTCGTCGCACTCGCCCGACGAGGAGT

Coding AGGCCRTCAAGGTCATGACGACGCTGGACATCCTCTCGTCGCACTCGCCCGACGAGGAGT

B73 AGGCCATCAAGGTCATGACGACGCTGGACATCCTCTCGTCGCACTCGCCCGACGAGGAGT

Va35 AGGCCATCAAGGTCATGACGACGCTGGACATCCTCTCGTCGCACTCGCCCGACGAGGAGT

T173 AGGCCATCAAGGTCATGACGACGCTGGACATCCTCTCGTCGCACTCGCCCGACGAGGAGT

Mp715 AGGCCATCAAGGTCATGACGACGCTGGACATCCTCTCGTCGCACTCGCCCGACGAGGAGT

Mp313E AGGCCATCAAGGTCATGACGACGCTGGACATCCTCTCGTCGCACTCGCCCGACGAGGAGT

27

Reference ACATGGGGGAGTTCGCGGAGCCGTCGTGGCTGGCGGAGCCCATGGTGAAGGCGGCGTTCG

Coding ACATGGGGGAGTTCGCRGAGCCRTCGTGGCTGGCGGAGCCCATGGTGAAGGCGGCGTTCG

B73 ACATGGGGGAGTTCGCGGAGCCGTCGTGGCTGGCGGAGCCCATGGTGAAGGCGGCGTTCG

Va35 ACATGGGGGAGTTCGCGGAGCCATCGTGGCTGGCGGAGCCCATGGTGAAGGCGGCGTTCG

T173 ACATGGGGGAGTTCGCGGAGCCGTCGTGGCTGGCGGAGCCCATGGTGAAGGCGGCGTTCG

Mp715 ACATGGGGGAGTTCGCGGAGCCGTCGTGGCTGGCGGAGCCCATGGTGAAGGCGGCGTTCG

Mp313E ACATGGGGGAGTTCGCGGAGCCGTCGTGGCTGGCGGAGCCCATGGTGAAGGCGGCGTTCG

Reference AGAAGTTCGGCGGCAGGATGAAGGAGATCGAGGGGTTCATCGACGAGTGCAACAACAACC

Coding AGAAGTTCGGCGGCAGGATGAAGGARATCGAGGGGTTCATCGACGAGTGCAACAACAACC

B73 AGAAGTTCGGCGGCAGGATGAAGGAGATCGAGGGGTTCATCGACGAGTGCAACAACAACC

Va35 AGAAGTTCGGCGGCAGGATGAAGGAGATCGAGGGGTTCATCGACGAGTGCAACAACAACC

T173 AGAAGTTCGGCGGCAGGATGAAGGAGATCGAGGGGTTCATCGACGAGTGCAACAACAACC

Mp715 AGAAGTTCGGCGGCAGGATGAAGGAGATCGAGGGGTTCATCGACGAGTGCAACAACAACC

Mp313E AGAAGTTCGGCGGCAGGATGAAGGAGATCGAGGGGTTCATCGACGAGTGCAACAACAACC

Reference TGGACCTCAAGAACCGCTGCGGCGCCGGGATCGTGCCGTACGAGCTGCTCAAGCCCTTCT

Coding TGGACCTCAAGAACCGCTGCGGMGCCGGGATCGTGCCGTACGAGCTGCTCAAGCCCTTCT

B73 TGGACCTCAAGAACCGCTGCGGCGCCGGGATCGTGCCGTACGAGCTGCTCAAGCCCTTCT

Va35 TGGACCTCAAGAACCGCTGCGGCGCCGGGATCGTGCCGTACGAGCTGCTCAAGCCCTTCT

T173 TGGACCTCAAGAACCGCTGCGGCGCCGGGATCGTGCCGTACGAGCTGCTCAAGCCCTTCT

Mp715 TGGACCTCAAGAACCGCTGCGGCGCCGGGATCGTGCCGTACGAGCTGCTCAAGCCCTTCT

Mp313E TGGACCTCAAGAACCGCTGCGGCGCCGGGATCGTGCCGTACGAGCTGCTCAAGCCCTTCT

Reference CCAAGCCGGGAGTCACCGGGAGGGGCATCCCCAGCAGCATCTCCATCTGATCCATCCTCA

Coding CCAAGCCGGGAGTCACCGGGAGGGGCATCCCCAGCAGCATCTCCATCTGATCCATCCTCA

B73 CCAAGCCGGGAGTCACCGGGAGGGGCATCCCCAGCAGCATCTCCATCTGATCCATCCTCA

Va35 CCAAGCCGGGAGTCACCGGGAGGGGCATCCCCAGCAGCATCTCCATCTGATCCATCCTCA

T173 CCAAGCCGGGAGTCACCGGGAGGGGCATCCCCAGCAGCATCTCCATCTGATCCATCCTCA

Mp715 CCAAGCCGGGAGTCACCGGGAGGGGCATCCCCAGCAGCATCTCCATCTGATCCATCCTCA

Mp313E CCAAGCCGGGAGTCACCGGGAGGGGCATCCCCAGCAGCATCTCCATCTGATCCATCCTCA

Reference GCATGCATTAGTCCAATTAA----ATCGGGGGGTGTACTATTATTGCATGCA----GAGG

Coding GCATGCATTAGTCYAATTAA----WTMGGGGGKTGTACTATTATTGCATGCA----GAGG

B73 GCATGCATTAGTCCAATTAA----ATCGGGGGGTGTACTATTATTGCATGCA----GAGG

Va35 GCATGCATTAGTCCAATTAATTAAATCGGGGGTTGTACTATTATTGCATGCA----GAGG

T173 GCATGCATTAGTCCAATTAATTAAATCGGGGGTTGTACTATTATTGCATGCA----GAGG

Mp715 GCATGCATTAGTCCAATTAATTAAATCGGGGGTTGTACTATTATTGCATGCA----GAGG

Mp313E GCATGCATTAGTCCAATTAA----ATCGGGGGTTGTACTATTATTGCATGCATGCAGAGG

Reference CTGCTTGTCGAATAAAACGTACTATATGTACGATTGTATTGTACATGTGTGTCAATGCAA

Coding CTGCTTGTCGAATAAAACGTACTATATGTAMGATTGTATTGTACAYGTGTGTYAATGCAA

B73 CTGCTTGTCGAATAAAACGTACTATATGTACGATTGTATTGTACATGTGTGTCAATGCAA

Va35 CTGCTTGTCGAATAAAACGTACTATATGTACGATTGTATTGTACATGTGTGTCAATGCAA

T173 CTGCTTGTCGAATAAAACGTACTATATGTACGATTGTATTGTACATGTGTGTCAATGCAA

Mp715 CTGCTTGTCGAATAAAACGTACTATATGTACGATTGTATTGTACATGTGTGTCAATGCAA

Mp313E CTGCTTGTCGAATAAAACGTACTATATGTACGATTGTATTGTACATGTGTGTCAATGCAA

Reference CAAGAGGCACGTTTGAGACTTTGAGTTAATACCAATTAAACTAGTAAGATGGCATTATTA

Coding CAAGAGGCACGTTTGAGACTTTGAGTTAATACCAATTAAMCTAGTAAGATGGMATTATTA

B73 CAAGAGGCACGTTTGAGACTTTGAGTTAATACCAATTAAACTAGTAAGATGGCATTATTA

Va35 CAAGAGGCACGTTTGAGACTTTGAGTTAATACCAATTAAACTAGTAAGATGGCATTATTA

T173 CAAGAGGCACGTTTGAGACTTTGAGTTAATACCAATTAAACTAGTAAGATGGCATTATTA

Mp715 CAAGAGGCACGTTTGAGACTTTGAGTTAATACCAATTAAACTAGTAAGATGGCATTATTA

Mp313E CAAGAGGCACGTTTGAGACTTTGAGTTAATACCAATTAAACTAGTAAGATGGCATTATTA

Reference ACAGAGCCTTTATACATTTTGAAACAACTTTATACATTTTGAAACGACTAGATATATATA

Coding ACAGAGCCTTTATACRTTTTGAAACAACTTTATACATTTTGAAAMSRMTAKATATATATA

B73 ACAGAGCCTTTATACATTTTGAAACAACTTTATACATTTTGAAACGACTAGATATATATA

Va35 ACAGAGCCTTTATACATTTTGAAACAACTTTATACATTTTGAAACGACTAGATATATATA

T173 ACAGAGCCTTTATACATTTTGAAACAACTTTATACATTTTGAAACGACTAGATATATATA

Mp715 ACAGAGCCTTTATACATTTTGAAACAACTTTATACATTTTGAAACGACTAGATATATATA

Mp313E ACAGAGCCTTTATACATTTTGAAACAACTTTATACATTTTGAAACGACTAGATATATATA

Reference TACAGTATAATTTTGAGATAGCTTCAAAGTACGAAAAGTAAAAATGACCCTGAAAGTGAT

Coding TACRR------------------------------------------------------T

B73 TACAG-------------------------------------------------------

Va35 TACAG-------------------------------------------------------

T173 TACAG-------------------------------------------------------

Mp715 TACAG-------------------------------------------------------

Mp313E TACAG-------------------------------------------------------

Reference AGAATA

Coding ATAATT

B73 ------

Va35 ------

T173 ------

Mp715 ------

Mp313E ------

Protein sequence alignment of GRMZM2G015419, *ZmLox10:*

CLUSTAL multiple sequence alignment by Kalign (2.0)

Blue Font= PS50095 PLAT Domain profile

Red Font= Lipoxygenase_3 domain: Lipoxygenase iron-binding catalytic domain

Dark red font (within the red font)= PS00711 Lipoxygenase_1: Lipoxygenases iron-binding regions signature 1

Purple font (within the red font)= PS00081 Lipoxygenase_2: Lipoxygenases iron-binding regions signature 2

Polymorphisms highlighted in yellow and are numbered as in Table 5 (only missense mutations from the DNA sequence).

3 5

Reference MMNLNLKQPLVLPAHHSNVVGSRLSSSSPSAAAASRRTGGGVSSRSGSRRHVRLPRISCS

B73 MMNLNLKQPLVLPAHHSNVVGSRLSSSSPSAAAASRRTGGGVSSRSGSRRHVRLPRISCS

Mp313E MMNLNLKQPLVLPAHHSNVVGSRLSSSSPSAAAASRRTGGGVSSRSGSRRHVRLPRISCS

Mp715 MMNLNLKQPLVLPAHHSNVVGSRLSSSSPSAAAASRRTGGGVSSRSGSRRHVRLPRISCS

T173 MMNLNLKQPLVLPAHHSNVVGSRLSSSSPSAAAASRRTGGGVSSRSGSRRHVRLPRISCS

Va35 MMNLNLKQPLVLPAHHSNVVGSRLSSPSPSAAAASRRTGGGVSSRSGSRRPVRLPRISCS

7 9

Reference ATEEVSGAVSSVTVERMLTVTASVEASPAIGQMYFQRAVDDIGDLLGKTLLLELVSSELD

B73 ATEEVSGAVSSVTVERMLTVTASVEASPAIGQMYFQRAVDDIGDLLGKTLLLELVSSELD

Mp313E ATEEVSGAVSSVTVERMLTVTASVEASPAIGQMYFQRAVDDVGDLFGKTLLLELVSSELD

Mp715 ATEEVSGAVSSVTVERMLTVTASVEASPAIGQMYFQRAVDDIGDLLGKTLLLELVSSELD

T173 ATEEVSGAVSSVTVERMLTVTASVEASPAIGQMYFQRAVDDIGDLLGKTLLLELVSSELD

Va35 ATEEVSGAVSSVTVERMLTVTASVEASPAIGQMYFQRAVDDVGDLFGKTLLLELVSSELD

13

Reference AKSGVEKTRVTAYAHKTLREGHYEAEFKVPASFGPVGAVLVENEHHKEVFIKEIKLVTGG

B73 AKSGVEKTRVTAYAHKTLREGHYEAEFKVPASFGPVGAVLVENEHHKEVFIKEIKLVTGG

Mp313E AKSGVEKTRVTAYAHKTLREGHYEAEFKVPASFGPVGAVLVENEHHKEVFIKEIKLVTGG

Mp715 AKSGVEKTRVTAYAHKTLREGHYEAEFKVPASFGPVGAVLVENEHHKEVFIKEIKLVTGG

T173 AKSGVEKTRVTAYAHKTLREGHYEAEFKVPASFGPVGAVLVENEHHKEVFIKEIKLVTGG

Va35 AKSGVEKTRVTAYAHKTLREGHYEAEFKVPASFGLVGAVLVENEHHKEVFIKEIKLVTGG

Reference DSSTAVTFDCNSWVHSKFDNPEKRIFFTLKSYLPSDTPKGLEDLRKKDLQALRGDGHGER

B73 DSSTAVTFDCNSWVHSKFDNPEKRIFFTLKSYLPSDTPKGLEDLRKKDLQALRGDGHGER

Mp313E DSSTAVTFDCNSWVHSKFDNPEKRIFFTLKSYLPSDTPKGLEDLRKKDLQALRGDGHGER

Mp715 DSSTAVTFDCNSWVHSKFDNPEKRIFFTLKSYLPSDTPKGLEDLRKKDLQALRGDGHGER

T173 DSSTAVTFDCNSWVHSKFDNPEKRIFFTLKSYLPSDTPKGLEDLRKKDLQALRGDGHGER

Va35 DSSTAVTFDCNSWVHSKFDNPEKRIFFTLKSYLPSDTPKGLEDLRKKDLQALRGDGHGER

14

Reference KVFERVYDYDVYNDLGDPDKNPAHQRPVLGGNKQYPYPRRCRTGRPRTKKDPETEMREGH

B73 KVFERVYDYDVYNDLGDPDKNPAHQRPVLGGNKQYPYPRRCRTGRPRTKKDPETEMREGH

Mp313E KVFERVYDYDVYNDLGDPDKNPAHQRPVLGGNKQYPYPRRCRTGRPRTKKDPETEMREGH

Mp715 KVFERVYDYDVYNDLGDPDKNPAHQRPVLGGNKQYPYPRRCRTGRPRTKKDPETEMREGH

T173 KVFERVYDYDVYNDLGDPDKNPAHQRPVLGGNKQYPYPRRCRTGRPRTKKDPETEMREGH

Va35 KVFERVYDYDVYNDLGDPDKNPAHQRPVLGGNKQYPYPRRCRTGRPRTKKDPETEMRKGH

15 16

Reference NYVPRDEQFSEVKQLTFGATTLRSGLHALLPALRPLLINKKDLRFPHFPAIDDLFSDGIP

B73 NYVPRDEQFSEVKQLTFGATTLRSGLHALLPALRPLLINKKDLRFPHFPAIDDLFSDGIP

Mp313E NYVPRDEQFSEVKQLTFGATTLRSGLHALLPALRPLLINKKDLRFPHFPAIDELYSDGIP

Mp715 NYVPRDEQFSEVKQLTFGATTLRSGLHALLPALRPLLINKKDLRFPHFPAIDDLFSDGIP

T173 NYVPRDEQFSEVKQLTFGATTLRSGLHALLPALRPLLINKKDLRFPHFPAIDELYSDGIP

Va35 NYVPRDEQFSEVKQLTFGATTLRSGLHALLPALRPLLINKKDLRFPHFPAIDELYSDGIP

17

Reference LPAQTGFDAIRTVVPRMVKLVEDTTDHVLRFEVPEMIERDRFSWFKDEEFARQTIAGLNP

B73 LPAQTGFDAIRTVVPRMVKLVEDTTDHVLRFEVPEMIERDRFSWFKDEEFARQTIAGLNP

Mp313E LPAQTGFDAIRTVVPRMVKLVEDTTDHVLRFEVPEMIERDRFSWFKDEEFARQTIAGLNP

Mp715 LPAQTGFDAIRTVVPRMVKLVEDTTDHVLRFEVPEMIERDRFSWFKDEEFARQTIAGLNP

T173 LPPQTGFDAIRTVVPRMVKLVEDTTDHVLRFEVPEMIERDRFSWFKDEEFARQTIAGLNP

Va35 LPAQTGFDAIRTVVPRMVKLVEDTTDHVLRFEVPEMIERDRFSWFKDEEFARQTIAGLNP

Reference LCIQLLTEFPIKSKLDPEVYGPAESAITKEILEKQMNGALTVEQALAAKRLFILDYHDVF

B73 LCIQLLTEFPIKSKLDPEVYGPAESAITKEILEKQMNGALTVEQALAAKRLFILDYHDVF

Mp313E LCIQLLTEFPIKSKLDPEVYGPAESAITKEILEKQMNGALTVEQALAAKRLFILDYHDVF

Mp715 LCIQLLTEFPIKSKLDPEVYGPAESAITKEILEKQMNGALTVEQALAAKRLFILDYHDVF

T173 LCIQLLTEFPIKSKLDPEVYGPAESAITKEILEKQMNGALTVEQALAAKRLFILDYHDVF

Va35 LCIQLLTEFPIKSKLDPEVYGPAESAITKEILEKQMNGALTVEQALAAKRLFILDYHDVF

Reference LPYVHKVRELQDATLYASRTIFFLTDLGTLMPLAIELTRPKSPTRPQWKRAFTHGPDATD

B73 LPYVHKVRELQDATLYASRTIFFLTDLGTLMPLAIELTRPKSPTRPQWKRAFTHGPDATD

Mp313E LPYVHKVRELQDATLYASRTIFFLTDLGTLMPLAIELTRPKSPTRPQWKRAFTHGPDATD

Mp715 LPYVHKVRELQDATLYASRTIFFLTDLGTLMPLAIELTRPKSPTRPQWKRAFTHGPDATD

T173 LPYVHKVRELQDATLYASRTIFFLTDLGTLMPLAIELTRPKSPTRPQWKRAFTHGPDATD

Va35 LPYVHKVRELQDATLYASRTIFFLTDLGTLMPLAIELTRPKSPTRPQWKRAFTHGPDATD

Reference AWLWKLAKAHVLTHDTGYHQLVSHWLRTHCCVEPYIIAANRQLSRLHPVYRLLHPHFRYT

B73 AWLWKLAKAHVLTHDTGYHQLVSHWLRTHCCVEPYIIAANRQLSRLHPVYRLLHPHFRYT

Mp313E AWLWKLAKAHVLTHDTGYHQLVSHWLRTHCCVEPYIIAANRQLSRLHPVYRLLHPHFRYT

Mp715 AWLWKLAKAHVLTHDTGYHQLVSHWLRTHCCVEPYIIAANRQLSRLHPVYRLLHPHFRYT

T173 AWLWKLAKAHVLTHDTGYHQLVSHWLRTHCCVEPYIIAANRQLSRLHPVYRLLHPHFRYT

Va35 AWLWKLAKAHVLTHDTGYHQLVSHWLRTHCCVEPYIIAANRQLSRLHPVYRLLHPHFRYT

23

Reference MEINALAREALINADGIIEESFWPGKYAVELSSVAYGATWQFDTEALPNDLIKRGLAVRG

B73 MEINALAREALINADGIIEESFWPGKYAVELSSVAYGATWQFDTEALPNDLIKRGLAVRG

Mp313E MEINALAREALINADGIIEESFWPGKYAVELSSVAYGATWQFDTEALPNDLIKRGLAVRG

Mp715 MEINALAREALINADGIIEESFWPGKYAVELSSVAYGATWQFDTEALPNDLVKRGLAVRG

T173 MEINALAREALINADGIIEESFWPGKYAVELSSVAYGATWQFDTEALPNDLVKRGLAVRG

Va35 MEINALAREALINADGIIEESFWPGKYAVELSSVAYGATWQFDTEALPNDLVKRGLAVRG

24

Reference EDGELELTIKDYPYAHDGLLVWDSIRQWASEYVNVYYKSDEAVAADPELRAFWDEVRNVG

B73 EDGELELTIKDYPYAHDGLLVWDSIRQWASEYVNVYYKSDEAVAADPELRAFWDEVRNVG

Mp313E EDGELELTIKDYPYAHDGLLVWDSIKQWASEYVNVYYKSDEAVAADPELRAFWDEVRNVG

Mp715 EDGELELTIKDYPYAHDGLLVWDSIKQWASEYVNVYYKSDEAVAADPELRAFWDEVRNVG

T173 EDGELELTIKDYPYAHDGLLVWDSIKQWASEYVNVYYKSDEAVAADPELRAFWDEVRNVG

Va35 EDGELELTIKDYPYAHDGLLVWDSIKQWASEYVNVYYKSDEAVAADPELRAFWDEVRNVG

Reference HGDKKDEPWWPVLDTRDSLVETLTTIMWVTSGHHSAVNFGQYHFAGYFPNRPTTIRKNMP

B73 HGDKKDEPWWPVLDTRDSLVETLTTIMWVTSGHHSAVNFGQYHFAGYFPNRPTTIRKNMP

Mp313E HGDKKDEPWWPVLDTRDSLVETLTTIMWVTSGHHSAVNFGQYHFAGYFPNRPTTIRKNMP

Mp715 HGDKKDEPWWPVLDTRDSLVETLTTIMWVTSGHHSAVNFGQYHFAGYFPNRPTTIRKNMP

T173 HGDKKDEPWWPVLDTRDSLVETLTTIMWVTSGHHSAVNFGQYHFAGYFPNRPTTIRKNMP

Va35 HGDKKDEPWWPVLDTRDSLVETLTTIMWVTSGHHSAVNFGQYHFAGYFPNRPTTIRKNMP

26

Reference VEEGGPGEEMEKFLKQPETTLLDMLPTQMQAIKVMTTLDILSSHSPDEEYMGEFAEPSWL

B73 VEEGGPGEEMEKFLKQPETTLLDMLPTQMQAIKVMTTLDILSSHSPDEEYMGEFAEPSWL

Mp313E VEEGGPGGEMEKFLKQPETTLLDMLPTQMQAIKVMTTLDILSSHSPDEEYMGEFAEPSWL

Mp715 VEEGGPGEEMEKFLKQPETTLLDMLPTQMQAIKVMTTLDILSSHSPDEEYMGEFAEPSWL

T173 VEEGGPGEEMEKFLKQPETTLLDMLPTQMQAIKVMTTLDILSSHSPDEEYMGEFAEPSWL

Va35 VEEGGPGGEMEKFLKQPETTLLDMLPTQMQAIKVMTTLDILSSHSPDEEYMGEFAEPSWL

Reference AEPMVKAAFEKFGGRMKEIEGFIDECNNNLDLKNRCGAGIVPYELLKPFSKPGVTGRGIP

B73 AEPMVKAAFEKFGGRMKEIEGFIDECNNNLDLKNRCGAGIVPYELLKPFSKPGVTGRGIP

Mp313E AEPMVKAAFEKFGGRMKEIEGFIDECNNNLDLRTAAAPD-RAVRAAQALLQAGVTGRGIP

Mp715 AEPMVKAAFEKFGGRR--------------------------------------------

T173 AEPMVKAAFEKFGGRMKEIEGFIDECNNNLDLKNRCGAGIVPYELLKPFSKPGVTGRGIP

Va35 AEPMVKAAFEKFGGRMKEIEGFIDECNNNLDLRTAAAPGSCR----TSCSSPSPSGSHRE

Reference SSISI

B73 SSISI

Mp313E SSISI

Mp715 -----

T173 SSISI

Va35 GHXQQHLHLIHPQHALVQLLNRGLYY---YCMQXLLVE
